# An Electrophysiological Index of Perceptual Goodness

**DOI:** 10.1093/cercor/bhw255

**Published:** 2016-12-26

**Authors:** Alexis D.J. Makin, Damien Wright, Giulia Rampone, Letizia Palumbo, Martin Guest, Rhiannon Sheehan, Helen Cleaver, Marco Bertamini

**Affiliations:** 1 Department of Psychological Sciences, University of Liverpool, Eleanor Rathbone Building, Liverpool, L69 7ZA, UK; 2 Liverpool Hope University, Hope Park, Liverpool, L16 9JD, UK

**Keywords:** symmetry, ERPs, sustained posterior negativity, holographic model, extrastriate cortex

## Abstract

A traditional line of work starting with the Gestalt school has shown that patterns vary in strength and salience; a difference in “Perceptual goodness.” The Holographic weight of evidence model quantifies goodness of visual regularities. The key formula states that *W = E/N*, where *E* is number of holographic identities in a pattern and *N* is number of elements. We tested whether *W* predicts the amplitude of the neural response to regularity in an extrastriate symmetry-sensitive network. We recorded an Event Related Potential (ERP) generated by symmetry called the Sustained Posterior Negativity (SPN). First, we reanalyzed the published work and found that *W* explained most variance in SPN amplitude. Then in four new studies, we confirmed specific predictions of the holographic model regarding 1) the differential effects of numerosity on reflection and repetition, 2) the similarity between reflection and Glass patterns, 3) multiple symmetries, and 4) symmetry and anti-symmetry. In all cases, the holographic approach predicted SPN amplitude remarkably well; particularly in an early window around 300–400 ms post stimulus onset. Although the holographic model was not conceived as a model of neural processing, it captures many details of the brain response to symmetry.

“Köhler was well aware and embarrassed by the circularity of the “law of Pragnanz”. Although his interests soon turned elsewhere, he never stopped hoping that a better definition would be found. When I took “the Köhler seminar” at Swarthmore in 1952, decades after Die Physischen Gestalten, one of the first tasks he put before us was to suggest definitions of “Pragnanz”. I don't recall that we had anything useful to say.”

Ulric Neisser (Biographical Memoirs, Volume 81, p.191)

## Introduction

Ernst Mach (1886/1959) observed that some types of symmetry are more obvious than others, while Gestalt psychologists considered the role of symmetry in perceptual organization (Wertheimer, 1923) and as a principle of pragnanz, or goodness of the stimulus (Kohler 1929; Koffka 1935). Perceptual goodness is related to response time or accuracy in symmetry discrimination tasks (e.g. Barlow and Reeves 1979; Royer 1981; Bertamini et al. 1997), but a formal definition has been problematic. The concept of perceptual goodness remains both fascinating and challenging (Kubovy and Wagemans 1995).

*Process models* of symmetry perception propose mechanisms that extract structure from the image (Wagemans et al. 1993; Dakin and Watt 1994). Goodness phenomena are attributed to different mechanisms and biases in the visual system. *Representational models,* in contrast, begin with abstract systems for coding information. For example, the *Transformational model* proposes that goodness increases with the number of structure-preserving transformations, such as the number of folds in a reflectional symmetry, the number of turns in a rotational symmetry, or the number of repeats in a repetition pattern (Garner 1974). However, the transformational model fails to explain why reflection has higher perceptual goodness than repetition or rotation, even when the number of transformations is matched.

The *Holographic Weight of Evidence model* is a representational model proposed by van der Helm and Leeuwenberg (1996), with roots in the Gestalt concept of perceptual economy (Attneave 1954; Garner 1962). There are 20 special *holographic regularities*: whenever they are subdivided into smaller pieces, all pieces have the same kind of regularity as each other, and as the original. Reflection, rotation, repetition, and Glass patterns (Glass 1969) have this holographic property.

The holographic model states that *W = E/N*: where *W* = perceptual goodness, *E* = evidence for regularity, and *N* = total amount of information. More precisely, *E* is the number of *holographic identities* in the pattern. Intuitively, *N* is total information and *E* is redundant information. The more information we “explain away” or “dismiss as a copy,” the larger the *E* term, and the higher the W-load of the pattern (van der Helm and Leeuwenberg 1996; Wagemans 1999).

*W* can be calculated in a straightforward way for dot patterns. For reflection with a single fold, *E* is number of pairs, and *N* is number of dots. This means that *W* = 0.5, however, many extra pairs are added. For Glass patterns, *E* is the number of co-oriented dipoles –1 and *N* is number of dots. *W* is slightly less than 0.5, but still largely independent of dot number. For repetition, *E* is the number of repeating blocks −1, and *N* is again number of dots. This means that *W* increases with number of blocks, but decreases with number of dots.

How can we test the Holographic model? Fechner envisaged two ways to discover laws relating the material and mental worlds: *Outer psychophysics* (plotting objective properties vs. subjective judgments) and *Inner psychophysics* (plotting objective properties vs. neurophysiology). Until now, the holographic model has only been tested with outer psychophysics, and it has been shown that W-load successfully predicts discrimination performance (Nucci and Wagemans 2007). Here, we moved to inner psychophysics for the first time: will W-load predict the brain response to symmetry?

The neural response to symmetry can be measured in different ways, as reviewed by Bertamini and Makin (2014). Functional magnetic resonance imaging (MRI) experiments have discovered symmetry activations in the form-sensitive Lateral-Occipital Complex (LOC), V4, and other extrastriate regions, but not in V1 or V2 (Sasaki et al. 2005, Tyler et al. 2005, Chen et al. 2007). Kohler et al. (2016) recently extended this by recording parametric activations to rotational symmetry in V3, V4 VO1 and LOC. Again, there was no symmetry response V1, or dorsal stream areas MT and IPS0. Furthermore, advanced electroencephalography (EEG) analysis showed that the ascending ventral areas were activated in temporal succession, with the symmetry response arriving V3 and V4 before LOC. Converging evidence comes from transcranial magnetic stimulation (TMS) studies, which have demonstrated a causal role for the LOC in symmetry perception (Bona et al. 2014, 2015).

ERP studies have consistently shown that amplitude at posterior electrodes is more negative for regular than random patterns from ~220 ms onwards (Norcia et al. 2002; Jacobsen and Höfel 2003; Höfel and Jacobsen 2007). This *Sustained Posterior Negativity* (SPN) is probably generated by the LOC and extrastriate cortex (Makin et al. 2012). The SPN can be generated by reflection, rotation or repetition, but it is largest for reflection (Makin et al. 2013). The SPN is similar whether people attend to symmetry or to other pattern features (Höfel and Jacobsen 2007; Makin et al. 2013, 2015).

After reviewing previous SPN recordings, we noticed that SPN amplitude was often larger for regularities with high perceptual goodness. We thus hypothesized that the holographic W-load would predict SPN amplitude.

## Previous Work

Before introducing our four new studies, we briefly revisit SPN recordings in earlier published work (Makin et al. 2012, 2013; Palumbo et al. 2015). All EEG experiments were conducted in the same laboratory using the same equipment, and analyzed in a similar way. We analyzed ERPs in posterior PO7/PO8 electrodes. The SPN was defined as the difference between regular and random waves from 300 to 1000 ms. Supplementary Material 1 provides a detailed explanation of the *W* calculation and EEG methods.

**Figure 1.**
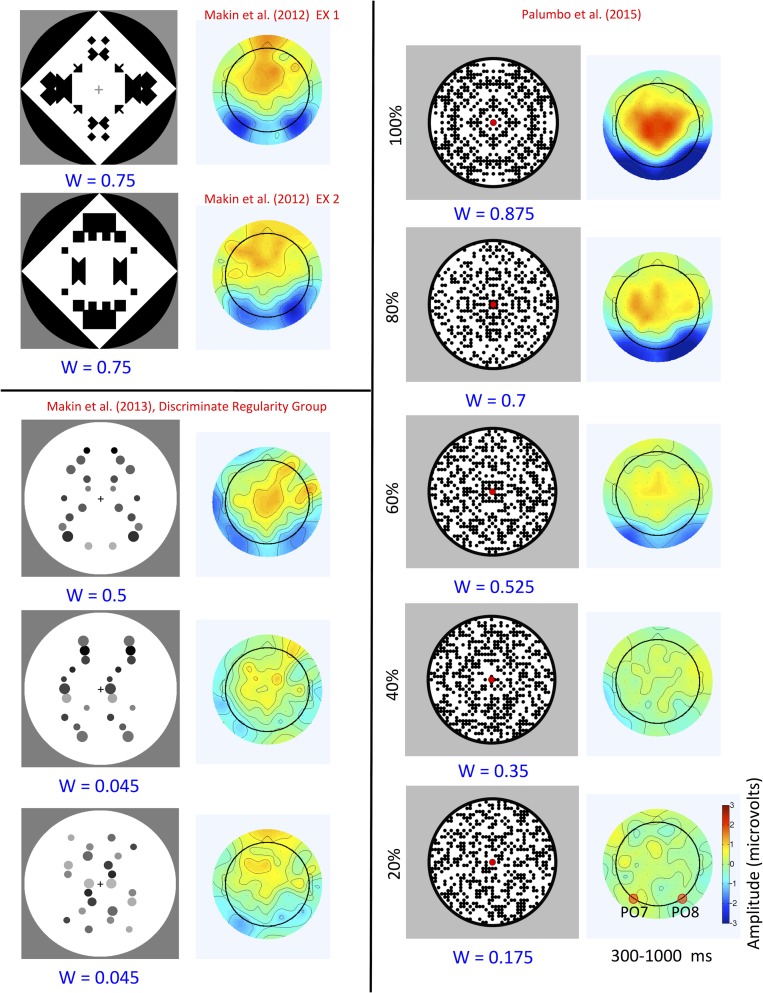
SPN scalp maps (regular–random, 300–1000 ms) and stimuli. Patterns with a larger *W*-load generate a larger SPN (blue at posterior electrodes).

**Figure 2.**
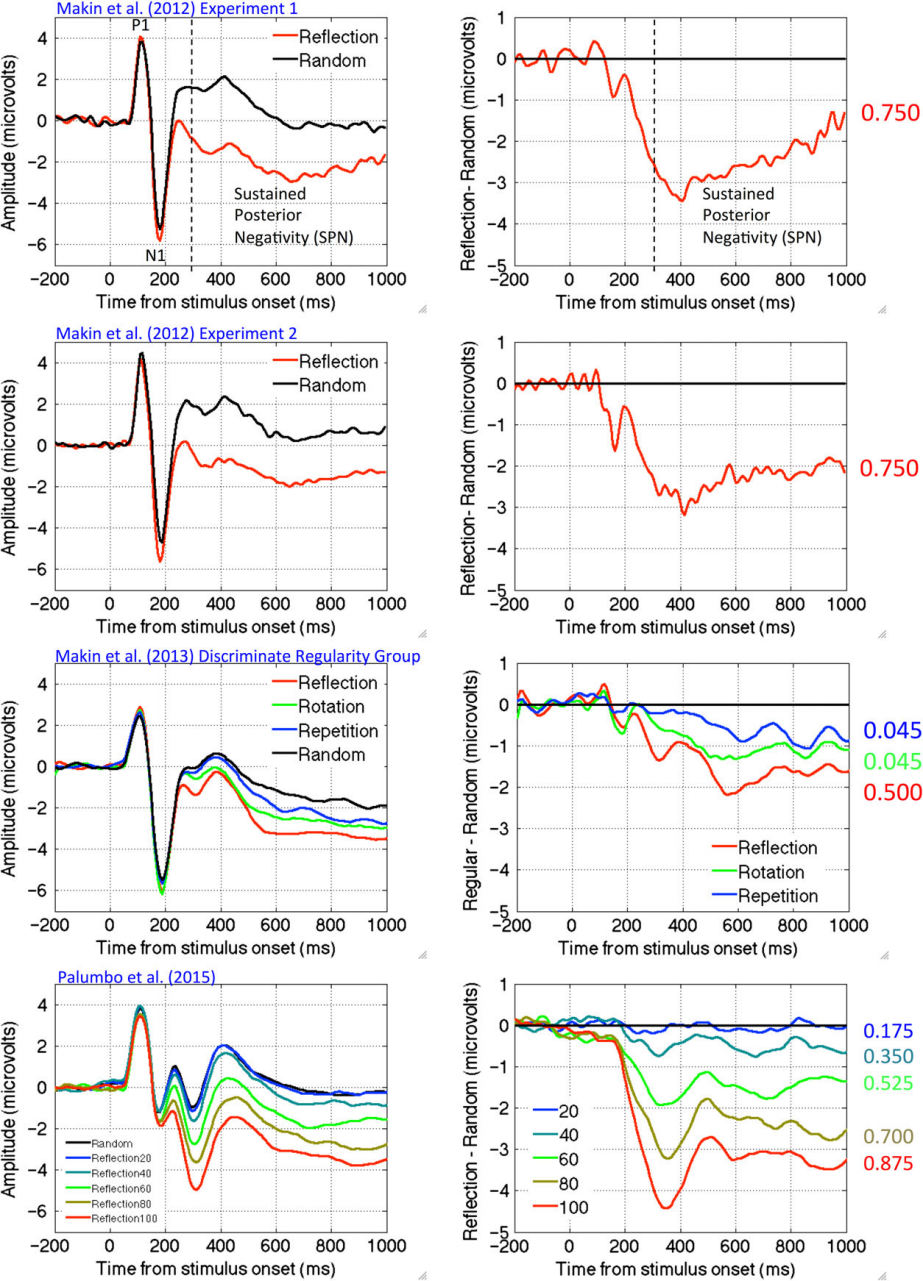
SPN waves from PO7/8 electrodes. Grand-average ERPs are shown in the left column, difference waves (regular–random) are shown in the right column. Colored numbers on the far right indicate the *W*-load of the patterns that generated these SPN waves.

Topographic difference maps are shown in Figure 1 (regular–random, 300–1000 ms) alongside the regular stimuli and their W-loads. The SPN is coded as *blue* at posterior electrodes. The higher W-load patterns generated larger SPN responses. Figure 2 shows the grand-average ERPs from PO7/8 and same data as difference wave (i.e. each regular ERP is subtracted from the random ERP). Again, we see that higher *W* patterns generated a larger (i.e. more negative) SPN wave.

**Figure 3.**
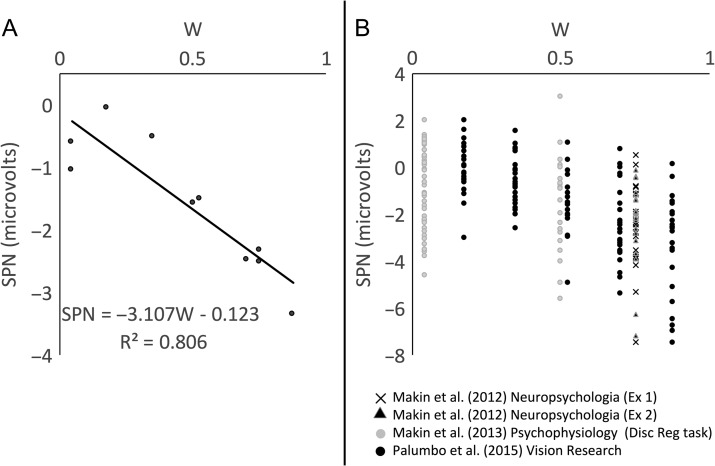
The relationship between *W*-load and SPN amplitude. (*A*) *W* versus grand-average SPN. Each datapoint is one grand-average SPN. (*B*) Same data, but with individual experiments and conditions grayscale-coded. Datapoints correspond to individual participants.

Figure 3*A* shows the linear relationship between *W* and SPN across all these experiments. W-load is plotted on the *x*-axis, and SPN amplitude on the *y*-axis. Each datapoint is a single grand-average SPN, from a particular condition of a particular experiment. Linear regression analysis of this data gives two interesting metrics: 1) the *slope* of the regression line (β), which shows how SPN amplitude changes with the *W*-load of the pattern and 2) the *fit* between regression line and the data (*R*^2^), which tells us the proportion of variance explained by *W*. Based on these data, we estimate that an increase in *W* from 0 to 1 produces a −3.107 μV change in SPN amplitude, and *W* explains ~81% of the variance in SPN amplitude (*R*^2^ = 0.806).

However, this basic regression analysis ignores the fact that datapoints are sub-conditions of different experiments with different samples. These secondary sources of variation are included in Figure 3*B*. We thus used more appropriate *linear mixed effects analysis* to assess statistical significance (lme4 library in R, Bates et al. 2015). W-load was entered as a fixed effect. The two random factors were Participant (slope and intercept) and Experiment (intercept). W-load had a significant effect on SPN amplitude (SPN (μV) = −3.396 W, χ^2^(1) = 46.362, *P* < 0.001).

In four new studies reported below, we obtained further estimates of the *W* versus SPN slope using linear mixed effects analysis. The mean slope estimate was −3.286. The standard deviation (SD) of all the slope estimates was just 0.386, even though stimuli and participants were all different (Table 1).

**Table 1:** Slope estimates for different measures of Perceptual goodness

	Min W	Max W	RT (s)	Error rate	SPN (uV)	GFP (uV)
Published	0.045	0.875	NA	NA	−3.396	1.138
Study 1	0.010	0.500	−1.106	−0.554	−2.855	0.614
Study 2	0.010	0.500	−1.226	−0.557	−3.069	0.706
Study 3	0.500	0.875	−0.467	−0.262	−3.875	1.308
All available	0.010	0.875	−0.939	−0.467	−3.234	0.932

Slopes were estimated with linear mixed effects analysis. Each row shows a particular data set (previously published, studies 1, 2, and 3, and all available data). Sustained Posterior Negativity (SPN) and Global Field Power (GFP) amplitudes were averaged 300–1000 ms window. Min and max *W* columns show the range of *W* values for stimuli included in the analysis.

## Study 1: *N* dots

The holographic model explains why the goodness of reflection is independent of dot number, while repetition becomes less obvious as we increase the number of dots. Conversely, the transformational approach to goodness (Garner 1974) does not predict these robust effects (van der Helm and Leeuwenberg 1996). We have already seen that reflection produces a larger SPN than repetition (Makin et al. 2013). Study 1 tests more detailed holographic predictions regarding *N* dots.

First, we report the results of a behavioral experiment where participants had to discriminate patterns as regular (either reflection or repetition) or random, as quickly and accurately as possible. Then we report ERPs generated by the same patterns, recorded from a different sample of participants.

## Study 1: Stimuli

**Figure 4.**
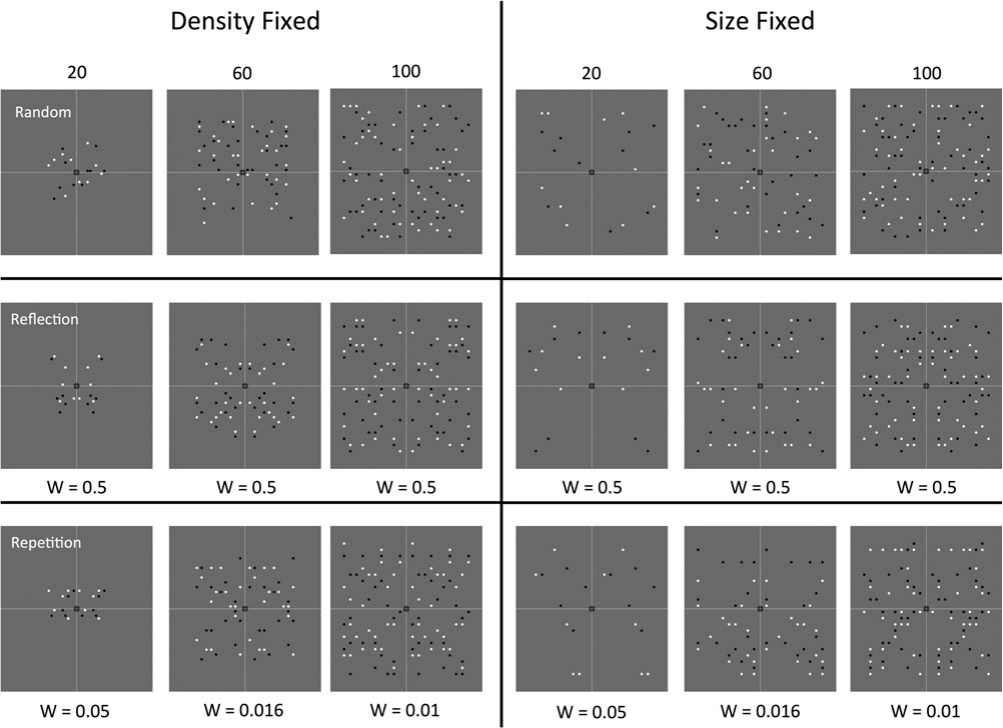
Study 1 stimuli and *W*-loads. The vertical and horizontal thin white lines were not present in the experiment. The central fixation dot was red.

Stimuli are shown in Figure 4. The largest patterns were ~5° wide. All patterns were presented on a gray background, and had an equal number of black and white elements, so overall luminance was not confounded with numerosity. The random patterns were constrained to have an equal number of elements on each side of the midline. It is impossible to vary *N* dots without varying either density or the size of the patterns. We controlled for this by including 50% density-fixed and 50% size-fixed trials. All analyses collapsed over this factor.

In both behavioral and EEG experiments, there were 720 trials in total. Half the trials were regular, half random. There were 60 repeats of each regular condition (Ref 20, Ref 60, Ref 100, Rep 20, Rep 60, and Rep 100) and 120 repeats of each random condition (Rand 20, Rand 60, and Rand 100).

## Study 1: Behavioral Experiment

**Figure 5.**
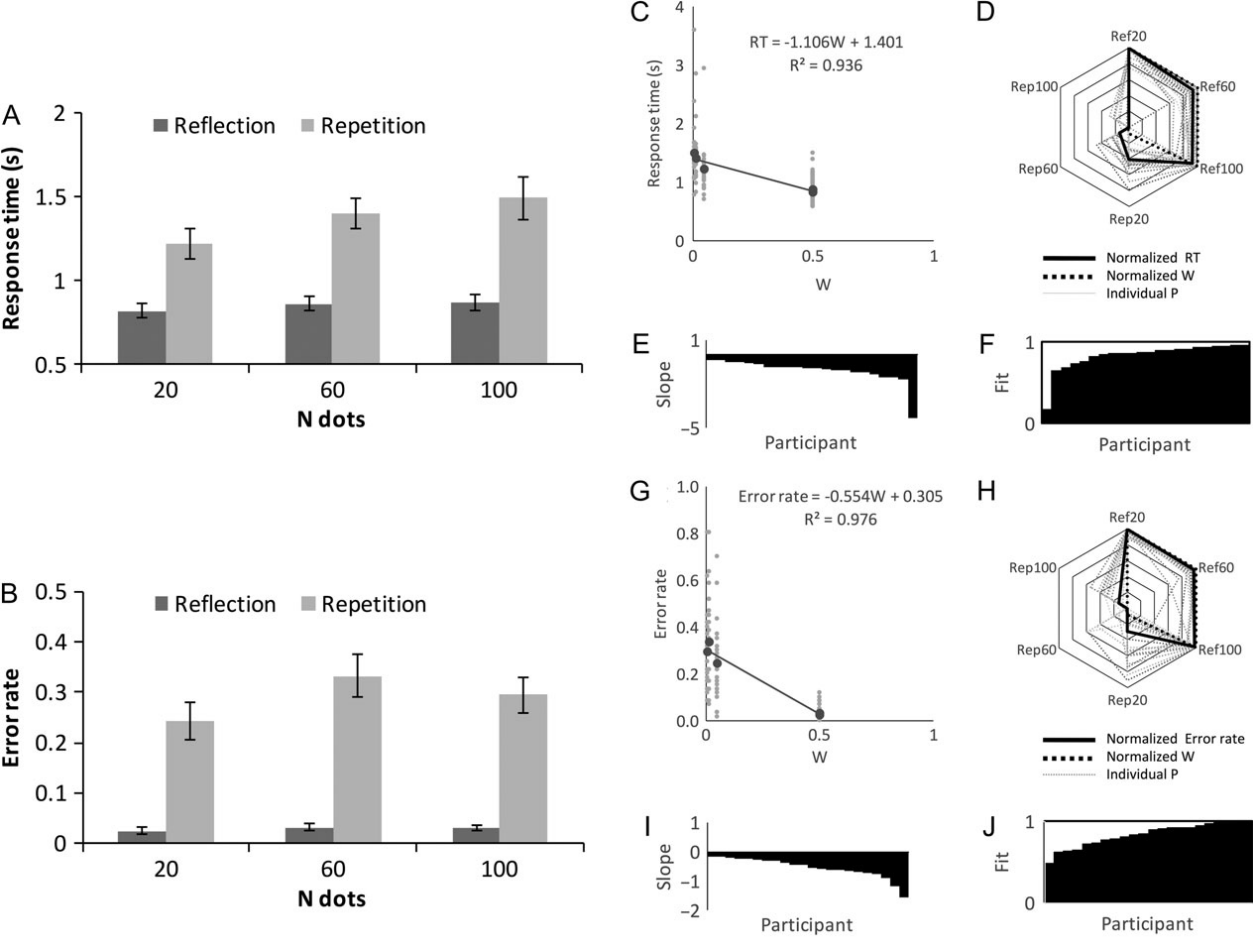
Study 1 behavioral results. Response time (*A*) and error rate (*B*) as a function of *N* dots in the reflection and repetition conditions. (*C*) Regression analysis of the *W* versus RT relationship (grand average in black, individual participants in gray, *R*^2^ indicates variance in *grand-average* RT explained by *W*). (*D*) Radar plot showing overlap between normalized *W* and RT. (*E*) Individual participant *W* versus RT slopes, organized cumulatively. (*F*) Individual participant *R*^2^, organized cumulatively. (*G–J*) Equivalent regression analysis on *W* versus Error rate.

A group of 22 participants (aged 18–32, 1 left-handed, 6 male) discriminated regular from random patterns as quickly and accurately as possible. Half the trials required a “Regular” response (left hand on “A” key) and half required a “Random” response (right hand on “L” key). Response time (RT) on the trials where participants pressed the correct button is shown in Figure 5*A*. In the repetition trials, RTs were long and increased with the number of dots. In reflection trials, RT was relatively short and independent of dot number.

Two-factor repeated measures ANOVA found main effects of Regularity (*F*_1,21_ = 40.599, *P* < 0.001, partial η^2^ = 0.659) and *N* dots (*F*_1.488__,__30.403_ = 27.246, *P* < 0.001, partial η^2^ = 0.565) and a Regularity×*N* dots interaction (*F*_1.555,32.662_ = 7.129, *P* = 0.005, partial η^2^ = 0.253). In the reflection condition, there was no effect of *N* dots on RT (*F*_1.380,28.983_ =2.360, *P* = 0.127). Conversely, in the repetition condition, RT increased with *N* dots (*F*_1.556,32.681_ = 18.548, *P* < 0.001, partial η^2^ = 0.469).

Error rate results were similar (Fig. 5*B*). There was again a main effect of Regularity (*F*_1,21_ = 56.235, *P* < 0.001, partial η^2^ = 0.728) and *N* dots (*F*_2,42_ = 8.295, *P* = 0.001, partial η^2^ = 0.283), and a Regularity×*N* dots interaction (*F*_2,42_ = 5.770, *P* = 0.006, partial η^2^ = 0.216). In the reflection condition, there was no effect of *N* dots on error rate (*F*_2,42_ = 0.715, *P* = 0.495). Conversely, in the repetition condition, error rate increased with when there were >20 dots (*F*_2,42_ = 7.556, *P* = 0.002, partial η^2^ = 0.265).

Next, we analyzed the relationship between *W* and RT with linear mixed effects analysis. *W* was the fixed effect. Participant slope and intercept were included as random factors. *W* was a significant predictor of RT (RT (s) = −1.106 W; χ^2^(1) = 23.920, *P* < 0.001). *W* explained most variance in grand-average RT (*R*^2^ = 0.936, Fig. 5*C*). The radar plot in Figure 5*D* illustrates the overlap between *normalized* RT and *W* (rectified and re-coded on a 0–1 scale). This scheme allows visualization of the similarity between *W* and RT: that is, we can see that *W* is closely related to RT because the *W* and RT contours are almost the same shape. Figure 5*E* and F shows slope and fit metrics from individual participants, organized cumulatively. This illustrates the consistency across the sample (mean *R*^2^ = 0.838).

Figure 5*G–J* shows equivalent analysis on Error rate. *W* was a significant predictor or Error rate (Error rate (*P*) = −0.554 *W*, χ^2^(1) = 28.475, *P* < 0.001). *W* captured most variance in grand-average error rate (*R*^2^ = 0.976). The same basic pattern was evident in all participants (mean *R*^2^ = 0.834).

## Study 1: ERP Experiment

Another 22 participants were involved in the ERP experiment (age 18–37, 4 left-handed, 11 male). Participants were required to fixate throughout both the 1.5 s baseline and 1.5 s stimulus presentation periods. Furthermore, they did not make a speeded response, but entered their judgment of “Regular” (i.e. either reflection or repetition) or “Random” after the stimulus disappeared (see Supplementary Material 2 for methods).

**Figure 6.**
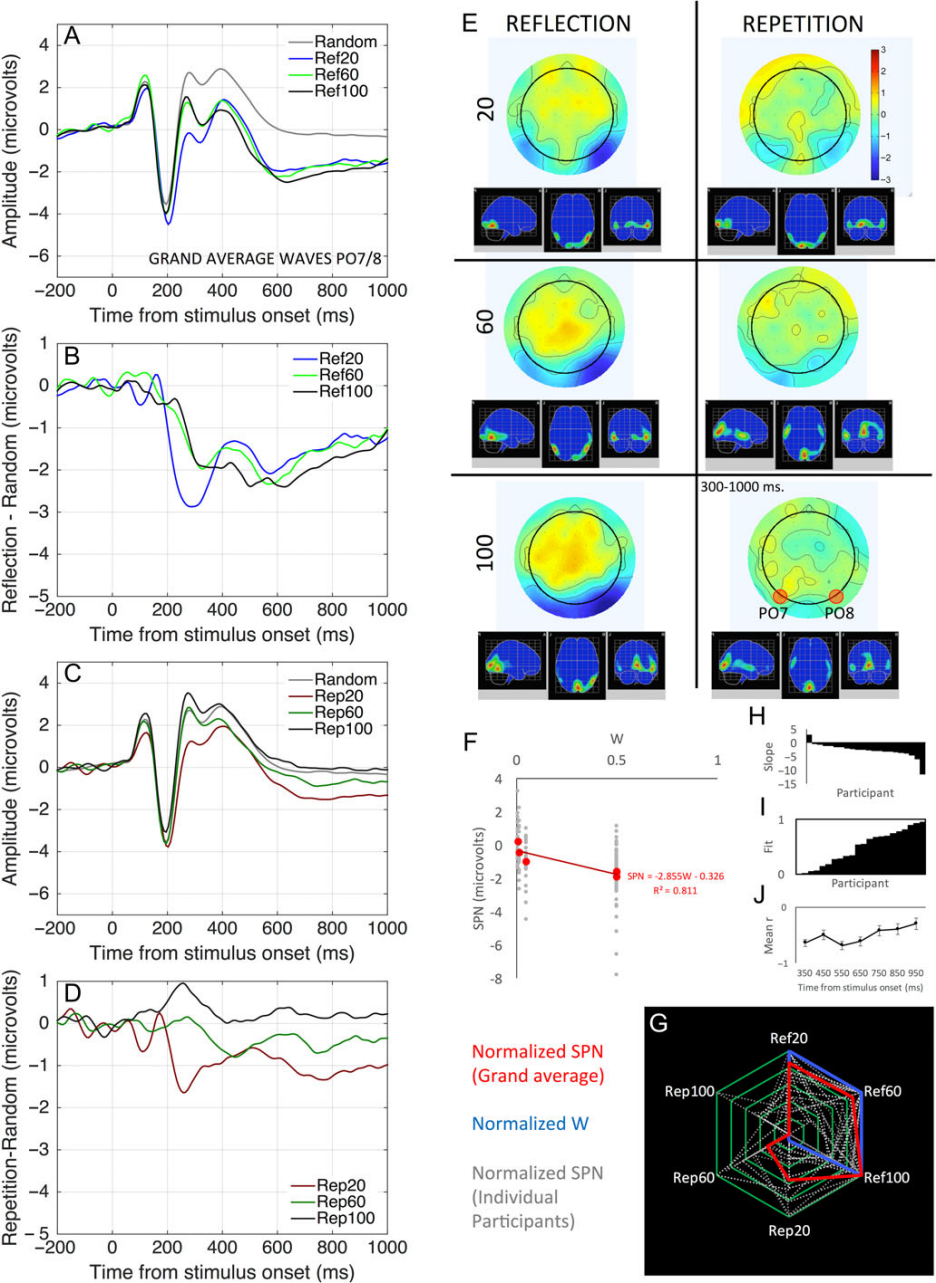
Study 1 ERP results. (*A–D*) Grand-average ERP waves at PO7/8 electrodes. (*E*) Topographic difference maps (regular–random, 300–1000 ms) and estimated cortical sources. (*F*) Regression analysis of *W* versus SPN relationship (grand average in red, individual participants in gray, *R*^2^ indicates the variance in *grand-average* SPN explained by *W*). (*G*) Radar plot showing overlap between normalized *W* and SPN (grand average in red, individual participants in gray). (*H*) Individual participant slope metrics, organized cumulatively. (*G*) Individual fit metrics, organized cumulatively. (*I*) Mean correlation between *W* and SPN in successive time bins. Error bars = ±1 SEM.

Grand-average ERPs from PO7/8 are shown in Figure 6*A–D*. As expected, reflection produced a larger SPN than repetition. For reflection, the SPN was independent of the number of dots. For repetition, the SPN amplitude declined with *N* dots.

Two-factor repeated measures ANOVA on SPN amplitude (300–1000 ms) found a main effect of Regularity (*F*_1,21_ = 25.711, *P* < 0.001, partial η^2^ = 0.550), but no main effect of *N* dots (*F*_2,42_ = 1.689, *P* = 0.197). There was a Regularity×*N* Dots interaction (*F*_2,42_ = 5.233, *P* = 0.009, partial η^2^ = 0.199), because there was a significant effect of *N* dots in the repetition condition (*F*_2,42_ = 4.944, *P* = 0.012, partial η^2^ = 0.191), but not in the reflection condition (*F*_2,42_ = 0.685, *P* = 0.509). One-sample *t*-tests show a significant SPN for all reflection patterns (i.e. regular–random <0, minimum effect, *t*(21) = −4.622, *P* < 0.001) and for the 20 dot repetition patterns (*t*(21) = −3.384, *P* = 0.003). There was no significant SPN in the 60 or 100 dot repetition trials (*t*(21) = −1.775, *P* = 0.090; *t* = 0.812, *P* = 0.426).

Figure 6*E* shows topographic difference maps for each condition (regular–random, 300–1000 ms) and an estimation of the cortical sources (computed with Low Resolution Electromagnetic Tomography, LORETA, Pascual-Marqui et al. 1994). The SPN was localized to bilateral posterior regions, broadly overlapping with the putative extrastriate symmetry network. This symmetry response was clearly stronger in the right hemisphere.

We note that ERP amplitude and topography are inevitably interconnected. Statistical topography analysis confirmed that while SPN amplitude varied with *W*, topography remained approximately constant (with some minor exceptions in the early part of the SPN time window, Supplementary Material 2).

Linear mixed effects analysis found that *W* was a significant predictor of SPN amplitude (SPN (μV) = −2.855 W, χ^2^(1) = 17.377, *P* < 0.001, Fig. 6*F*), and there was the high overlap between grand-average SPN and *W* (*R*^2^ = 0.811, Fig. 6*G*). The same effect was present in 21/22 participants (mean *R*^2^ = 0.484, with negative slopes coded as *R*^2^ = 0, Fig. 6H and *I*).

We typically treat SPN amplitude as a single number (regular–random, 300–1000 ms, at PO7/8). To assess evolution of the symmetry *within* the 300–1000 ms time window, we measured the *W* versus SPN correlation in seven successive 100 ms time bins. The correlation declined after ~700 ms (Fig. 6*J*). Additional analysis of the SPN evolution is included in Supplementary Material 2. *W* generally explained more variance in SPN amplitude during the early part of the interval, but still captures some SPN variance at the end as well.

Finally, we explored *Global Field Power* (GFP) of the regular–random difference maps. GFP is the SD of amplitudes across the 64 electrodes at a particular time point. The more color variation there is in a topographic map, the higher the GFP. As described in Supplementary Material 2, *W* explained most variance in average GFP. This increases confidence that our results are not problematically dependent on the electrodes selected for SPN analysis.

## Study 1: Discussion

Study 1 confirmed the predictions from the holographic model. As expected, the SPN was larger for reflection than for repetition, and only sensitive to *N* dots for repetition. The hypothesized behavioral results were also found, broadly replicating previous work (Csathó et al. 2003).

In Study 1, *W* explained ~81% of variance in grand-average SPN, and a change in *W* from 0 to 1 produces a −2.855 μV change in SPN amplitude. This is comparable to *W* versus SPN slope estimates based on other data sets, albeit slightly lower (Table 1).

More fine-grained analysis revealed several important features. First, *W* was most closely related to SPN amplitude in the early part of the window, although there was a relationship throughout. Second, the SPN was consistently source-localized to extrastriate visual areas, predominantly in the right hemisphere. This is consistent with previous SPN source-localization analysis (Makin et al. 2012) and with fMRI results (e.g. Sasaki et al. 2005). A right hemisphere advantage for symmetry processing has also be found with TMS and occipital alpha desynchronization analysis (Verma et al. 2013; Bona et al. 2014; Wright et al. 2015).

To control luminance, all patterns had 50% black and 50% white dots. However, participants may have selectively attended to half the dots (either black or white). This could effectively *double* the *W*-load of the repetition patterns by *halving* *N*. This could explain why the SPN for repetition patterns was larger than the holographic model predicted.

## Study 2: Glass Patterns, Reflection, and Repetition

Study 2 tested the holographic model predictions regarding reflection, repetition, and concentric Glass patterns. As with Study 1, we ran complementary behavioral and EEG experiments on different samples. All patterns had 100 dots. *W*-load was 0.5 for reflection, 0.49 for Glass, but only 0.01 for repetition. We thus predicted that the SPN would be approximately the same amplitude for reflection and Glass patterns, but much reduced for repetition.

## Study 2: Stimuli

**Figure 7.**
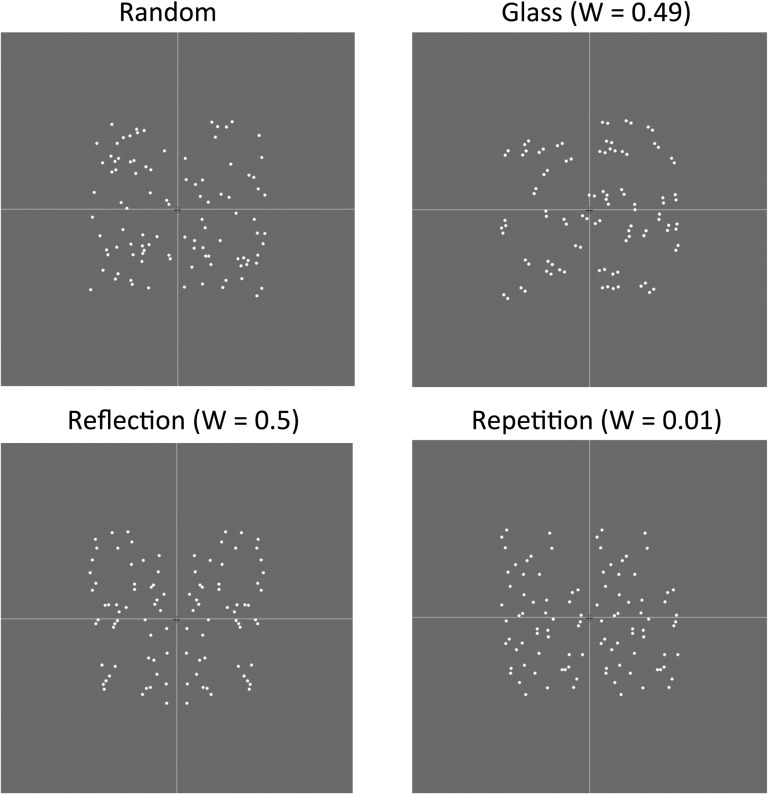
Study 2 stimuli and *W*-loads. The thin white lines were not present in the experiment.

Stimuli are shown in Figure 7. All patterns were on a gray background and dots fell within an ~5×5° area in the center of the screen. There were 100 dots in all patterns, although for the Glass patterns these were organized into 50 dipoles. Dots in the dipoles were always 0.15° from each other, irrespective of eccentricity. This was consistent with recent neuropsychological experiments using concentric Glass patterns (Lestou et al. 2015). All dots were white, allowing unambiguous calculation of *W*. The random patterns were constrained to have an equal number of elements on each side of the midline. There were 360 trials in total, with 60 repeats of each regular condition (Glass, reflection, and repetition) and 180 random trials.

## Study 2: Behavioral Experiment

A group of 22 participants (age 18–51, 1 left-handed, 11 male) discriminated regular (Glass, reflection, or repetition) from random patterns as quickly and accurately as possible.

**Figure 8.**
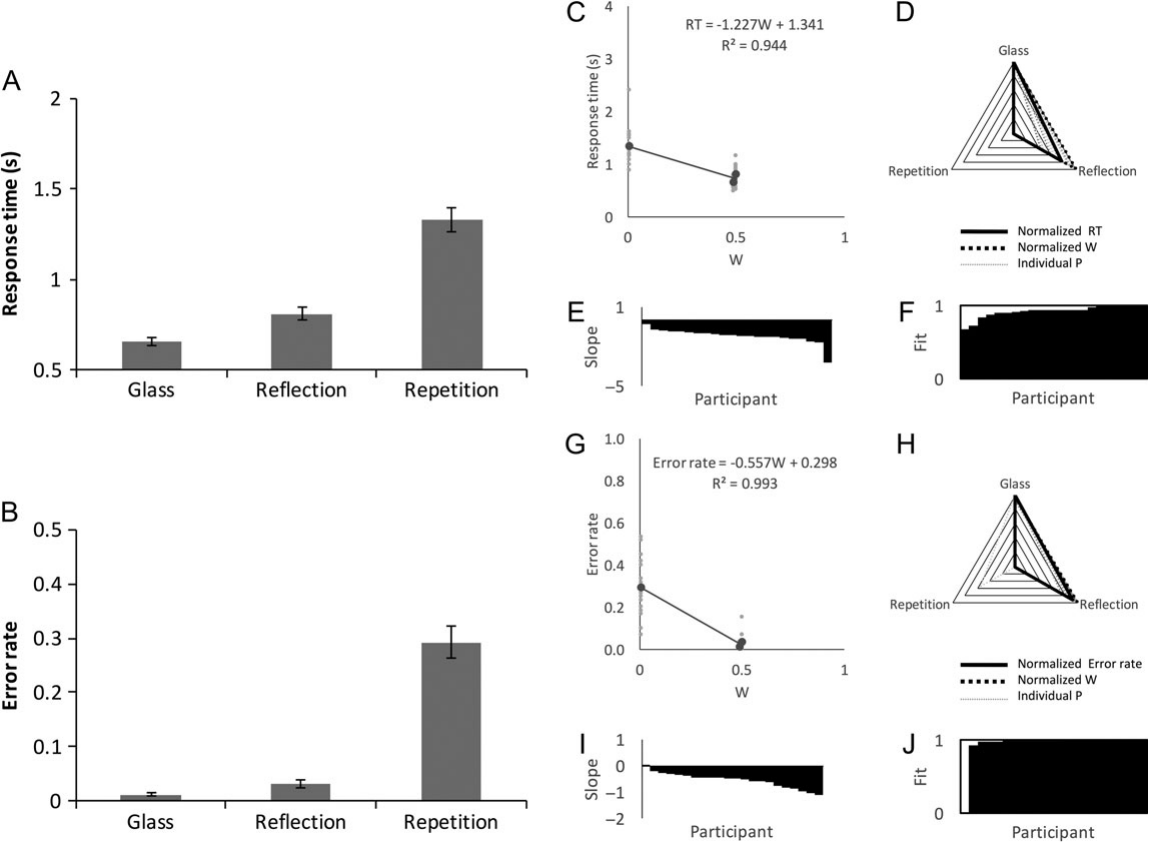
Study 2 behavioral results. Conventions are the same as Figure 5. (*A*) Response times in Glass, reflection and repetition conditions. (*B*) Error rate. (*C*) Regression analysis of the *W* vs. RT relationship. (*D*) Overlap between normalized *W* and RT. (*E*) Individual participant W vs. RT slopes, organized cumulatively. (*F*) Individual fit metrics, organized cumulatively. (*G-J*) Equivalent regression analysis on W vs. Error rate.

RT results are shown in Figure 8*A*. There was a main effect of Regularity (*F*_1.210,25.420_ = 102.695, *P* < 0.001, η^2^ = 0.830). Participants took longer to classify repetition than reflection (*t*(21) = 9.117, *P* < 0.001) or Glass patterns (*t*(21) = 11.288, *P* < 0.001). Unexpectedly, participants were also slightly faster to classify Glass patterns than reflection (*t*(21) = 7.113, *P* < 0.001).

Error rate results were similar to RT results (Fig. 8*B*). There was a main effect of Regularity (*F*_1.110, 23.315_ = 83.417, *P* < 0.001, η^2^ = 799). Error rate was higher for repetition than for reflection (*t*(21) = 8.586, *P* <0.001) or Glass patterns (*t*(21) = 10.074, *P* < 0.001). Error rate was also higher for reflection than for Glass patterns (*t*(21) = 2.581, *P* = 0.017).

Figure 8*C–F* illustrates the relationship between *W* and RT. Linear mixed effects analysis confirmed that *W* was a significant predictor of RT (RT(s) = −1.227 W, χ^2^(1) = 39.875, *P* < 0.001). Fit between model and RT was near perfect (*R*^2^ = 0.944), and the same effects were found in all 22 participants (mean *R*^2^ = 0.923).

The error rate results are shown in Figure 8*G–J*. *W* was a significant predictor of error rate (error rate (*P*) = −0.557 W, χ^2^(1) = 36.072, *P *< 0.001). *W* explained nearly all variance in grand-average error rate (*R*^2^ = 0.993), and comparable effects were found in 21/22 participants (mean *R*^2^ = 0.945).

## Study 2: ERP Experiment

**Figure 9.**
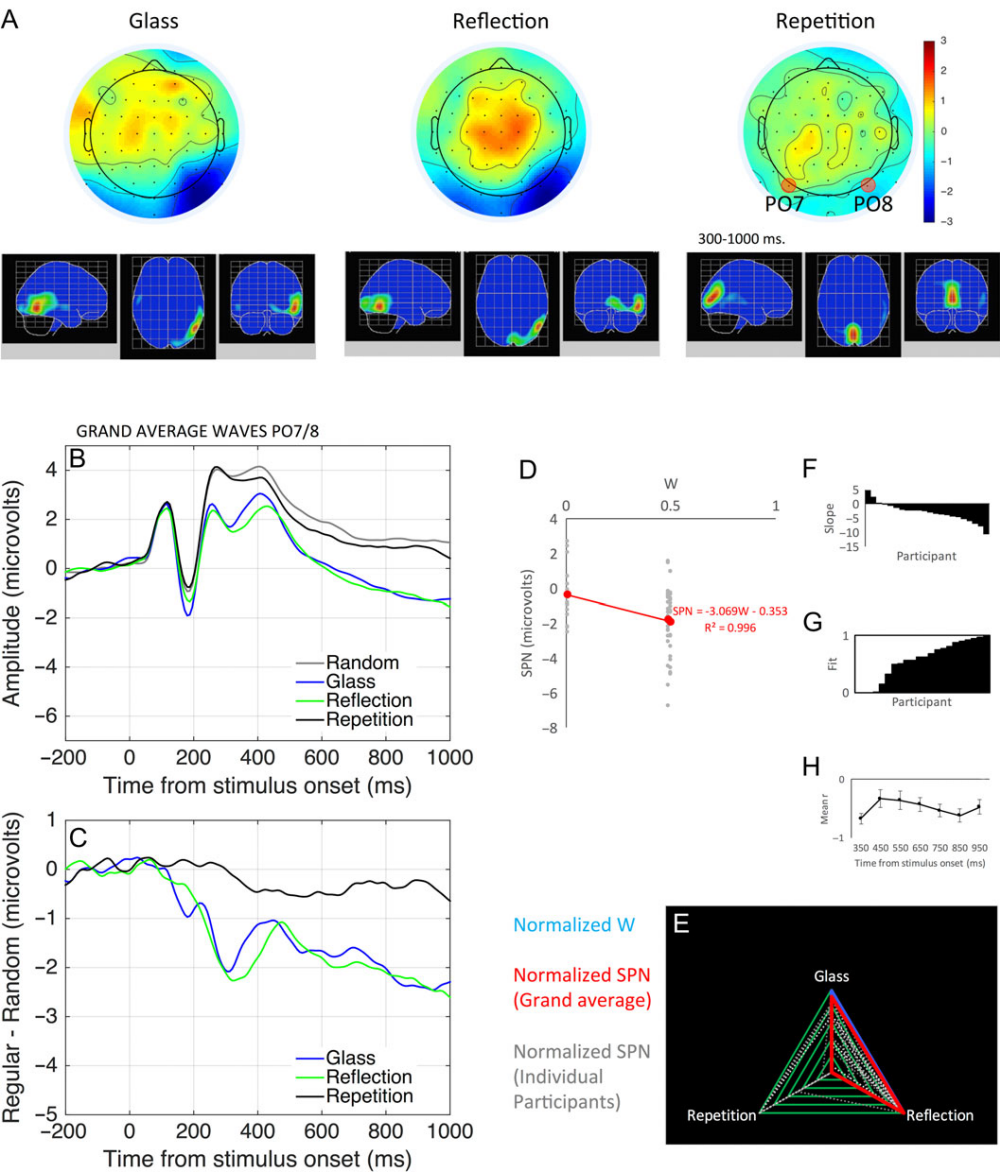
Study 2 ERP results. Conventions are the same as Figure 6. (*A*) Topographic difference maps (regular -- random) and estimated cortical sources. (*B*) Grand-average ERP waves at PO7/8 electrodes. (*C*) Regular -- random difference waves. (*D*) Regression analysis of *W* vs. SPN relationship. (*E*) Overlap between normalized W and SPN. (*F*) Individual participant slope metrics, organized cumulatively. (*G*) Individual fit metrics, organized cumulatively. (*H*) Mean correlation between W and SPN in successive time bins.

Another 22 participants were involved in the EEG experiment (age 18–33, 2 left-handed, 10 male). Methods are described in Supplementary Material 3. Figure 9*A* shows topographic difference plots and estimated anatomical location of the cortical generators. Reflection and Glass patterns activated the same posterior brain regions, predominantly in the right hemisphere. Statistical topography analysis confirmed that reflection and Glass topographies were similar (Supplementary Material 3).

Grand-average ERPs from PO7/8 and difference waves are shown in Figure 9*B* and C. As expected, reflection and Glass patterns produced a comparable SPN, while repetition produced a weaker response.

SPN data (300–1000 ms) were analyzed with repeated measures ANOVA. There was a significant main effect of Regularity (*F*_2,42_ = 7.538, *P *= 0.002, η^2^ = 0.264). Both Glass and reflection differed from repetition (*t*(21) = −3.492, *P *= 0.002, *t*(21) = −3.519, = 0.002), but not from each other (*t*(21) = 0.299, *P *= 0.768). There was a significant SPN for Glass (*t*(21) = 4.256, *P *< 0.001) and reflection (*t*(21) = 5.188, *P *< 0.001), but not for repetition (*t*(21) = 1.292, *P *= 0.210).

*W* was a significant predictor of SPN amplitude (SPN (μV) = −3.069 W; χ^2^(1) = 12.414, *P *< 0.001, Figure 9*D–G*), which explained most variance in grand-average SPN (*R*^2^ = 0.996). There was a similar effect in 19/22 participants (mean *R*^2^ = 0.566). Figure 9*H* shows that the negative *W* versus SPN correlation was largest at 300–400 ms (see Supplementary Material 3 for more analysis of temporal evolution and consistent GFP analysis).

## Study 2: Discussion

Study 2 supported the holographic model. The SPN waves generated by reflection and Glass patterns were similar, and larger than repetition. Reflection and Glass patterns produced similar topographic maps, probably generated by right extrastriate networks. The *W* versus SPN slopes were consistent with other studies (Table 1). However, *R*^2^ was probably overestimated in Study 2, because we were fitting a regression line to just three datapoints. Finally, the holographic model explained SPN data well throughout the SPN window, with some evidence for an early peak.

We warn that these results may be specific to *concentric* Glass patterns. Our preliminary data show a reduced SPN for *translational* Glass patterns, while neuroimaging work has shown differences between concentric and translational configurations (Lestou et al. 2015). This is a topic for future research.

## Study 3: *N*-Folds

The holographic model is not the only representational account of symmetry. In Study 3, we contrasted the predictions of the holographic model with the predictions of the transformational model (Garner 1974).

To understand the transformational model, consider a reflectional symmetry pattern with single axis: We can rigidly rotate it 180° in depth around the axis, and structure is preserved. This is an example of *invariance under transformation*. If we add another fold, then another invariance transformation is added to the pattern. It could be that perceptual goodness scales with the number of rigid transformations in the pattern. Interestingly, the holographic and transformational models make different predictions about how goodness should increase with the number of folds in reflection (van der Helm and Leeuwenberg 1996, van der Helm 2011).

Van der Helm (2011) quantified the predictions of the transformational model. We denote these transformational predictions as *T*. According to van der Helm (2011), *T* = 1−(1/2 *F*), where *F* is the number of folds. Study 3 examined reflectional symmetry with 1 to 5 folds. We tested whether the SPN would be more closely related to *W* (from the holographic model) or *T* (from the transformational model).

## Study 3: Stimuli

**Figure 10.**
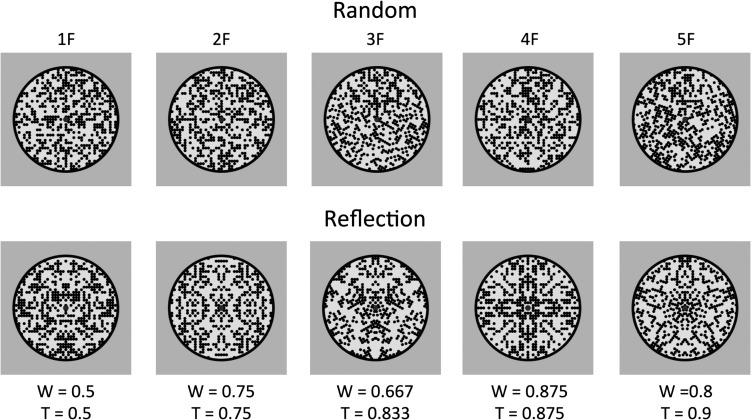
Study 3 stimuli. The predicted goodness levels from the holographic (*W*) and transformational (*T*) model are indicated below the *N*-fold reflections.

Example stimuli with the corresponding *W* and T scores are shown in Figure 10. Stimuli construction was similar to that described in Palumbo et al. (2015). There were around 510–540 dots in each pattern. The mean number of dots varied slightly with *N* axis. The SD of *N* dots also increased with the number of axes from approximately 16 dots in the random conditions to around 50 in the 5-fold condition. On each trial, the program constructed the patterns by first making a single grid segment, and randomly deciding which positions on the grid would be filled. We set a 40% probability of occupation. More folds meant smaller segments, and consequently greater deviations from the mean of 40% occupancy. We think it is very unlikely that these small confounds had a substantial effect on ERPs or behavioral responses. There were 60 repeats of each condition, giving 600 trials in total (300 reflections and 300 random).

## Study 3: Behavioral Experiment

**Figure 11.**
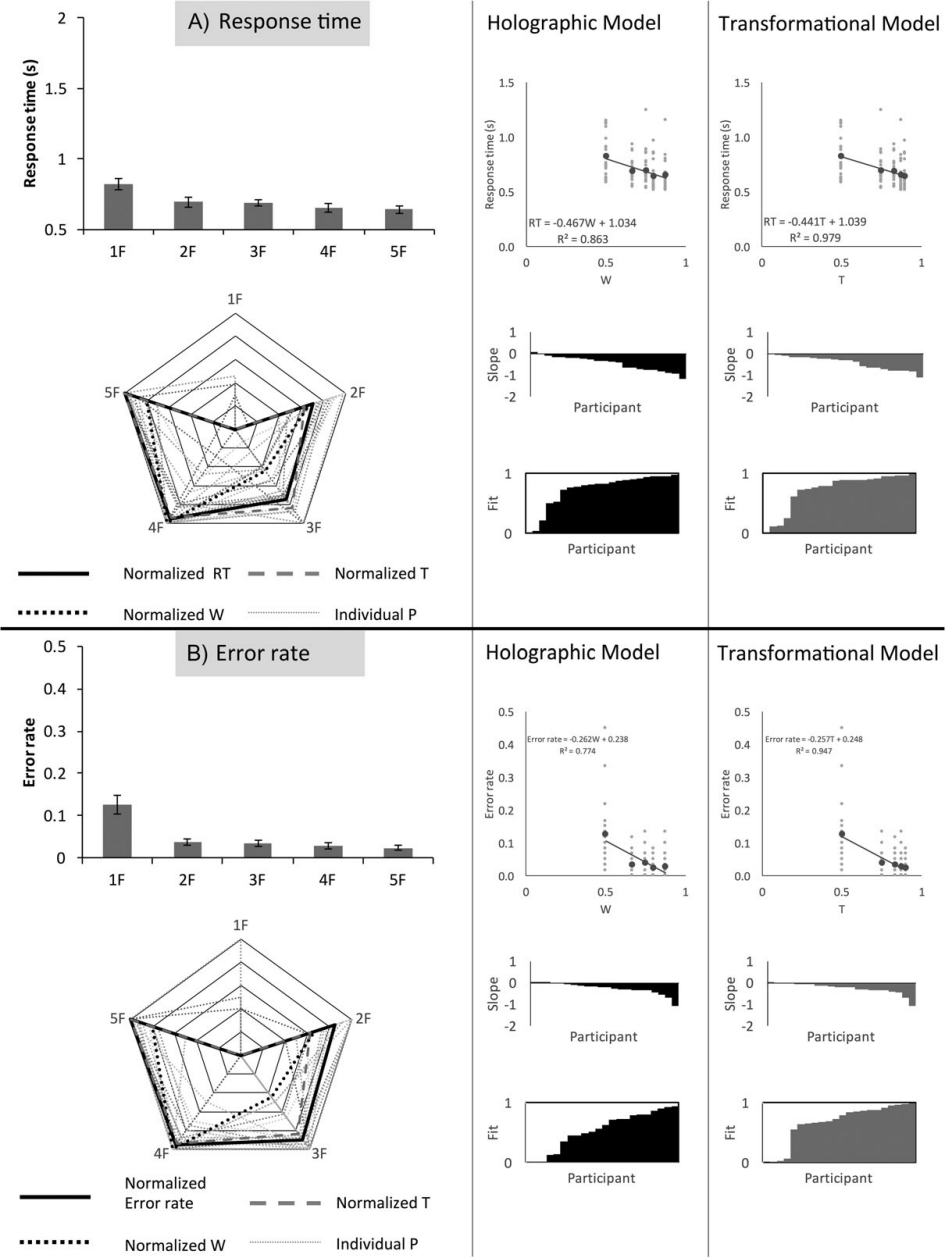
Study 3 behavioral results. (*A*) Response times. (*B*) Error rate. Conventions are the same as Figure 5, although both holographic (*W*) and transformational (*T*) predictions are included.

In the behavioral experiment, 22 participants (age 20–54, 1 left-handed, 9 male) discriminated regular from random patterns as quickly and accurately as possible. RT results are shown in Figure 11*A*. There was a main effect of folds (*F*_2.049, 43.024_ = 27.457, *P *< 0.001, η^2^ = 0.567). There was a significant reduction in RT from 1-fold to 2-fold (*t*(21) = 4.796, *P *< 0.001), and from 3-fold to 4-fold (*t*(21) = 2.352, *P *= 0.029).

Figure 11*A* illustrates the fit between normalized RT data, *W* and T. The five grand-average RTs were similar to normalized *T* (*R*^2^ = 0.979) and less similar to normalized *W* (*R*^2^ = 0.863). Both *W* and T were significant predictors of RT (RT = −0.467 *W*, χ^2^(1) = 24.319, *P *< 0.001; RT = −0.441 T, χ^2^(1) = 25.401, *P *< 0.001).

Next, we conducted another mixed effects analysis using *residuals* of the *T* versus *W* regression line as a fixed effect. This analysis tested whether the *difference between* *W* and *T* explained any variance in RT (as we would expect, if one model was better than the other). This analysis revealed that *T* predicted more variance in RT than *W* (χ^2^(1) = 10.961, *P *< 0.001). However, model fits were comparable at an individual participant level (mean *R*^2^ = 0.724 vs. 0.728, *t*(21) = −0.131, *P *= 0.897).

Error rate results are shown in Figure 11*B*. The effect of folds on error rate was significant (*F*_1.424, 29.895_ = 18.919, *P *< 0.001, η^2^ = 0.474). There was a significant reduction in error from 1- to 2-fold only (*t*(21) = 4.456, *P *< 0.001). Again there was very close overlap between the five grand-average error rates and *T* (*R*^2^ = 0.947), and less overlap between five grand-average error rates and *W* (*R*^2^ = 0.774). Both *W* and *T* predicted error rate (error rate (*P*) = −0.262 W, χ^2^(1) = 15.417, *P* < 0.001; error rate (*P*) = −0.257 T, χ^2^(1) = 16.336, *P* < 0.001), but *T* explained more variance in error rate than *W* (χ^2^(1) = 13.315, *P* < 0.001). Likewise, at an individual participant level, mean *R*^2^ was greater for *T* than *W* (0.663 vs. 0.542, *t*(21) = 3.289, *P* = 0.003).

## Study 3: ERP Experiment

Another 22 participants were involved in the EEG experiment (age 18–45, 5 left-handed, 5 male, see Supplementary Material 4 for details).

**Figure 12.**
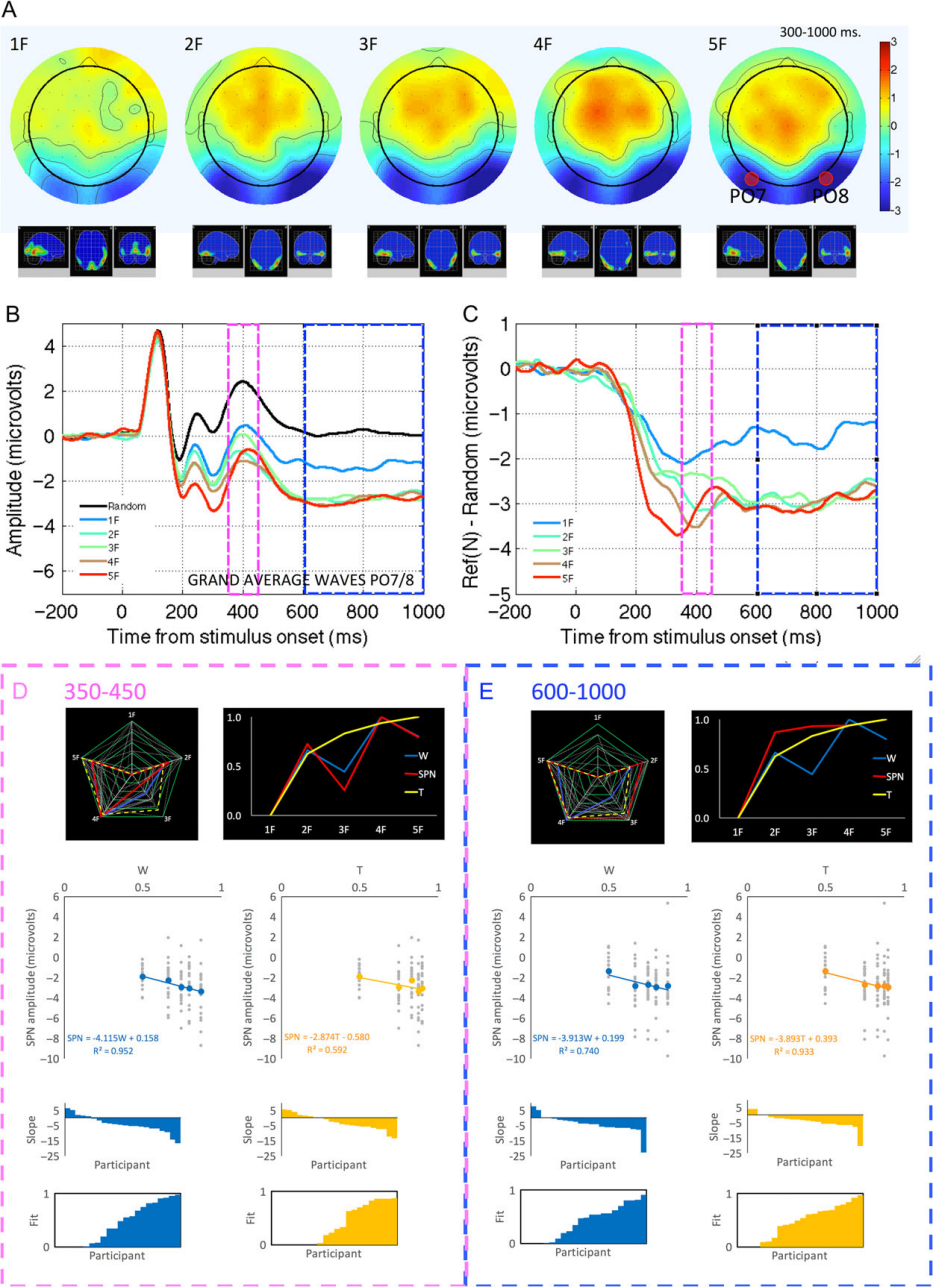
Study 3 ERP results. (*A*) Topographic difference maps and estimated cortical sources. (*B*) Grand-average ERP waves at PO7/8 electrodes. (*C*) Reflection -- random difference waves. The early (350--450 ms) and late (600--1000 ms) windows are highlighted (blue and pink boxes). (*D*) Analysis of the early window. (*E*) Analysis of the late window.

Topographic difference maps are shown in Figure 12*A*. All patterns produced an SPN, localized to the bilateral extrastriate visual cortex, with no right lateralization. SPN amplitude was clearly reduced for the 1-fold patterns. SPN waves for the multiple symmetries did not line up consistently. Supplementary Material 4 shows that SPN topography was largely invariant, while amplitude changed with *N*-folds.

As with the other studies, we also considered SPN evolution across the 300–1000 ms window (Supplementary Material 4). *W* was particularly closely related to SPN at around 350–450 ms. This corresponded to a discrete positive deflection in the ERP waveforms (Fig. 12*B* and *C*, pink boxes), while *T* was more closely related to SPN amplitude later on, at around 600–1000 ms (Fig. 12*B* and *C*, blue boxes). We thus move straight into exploratory analysis of these early and late windows (rather than first analyzing the traditional 300–1000 ms SPN data).

SPN amplitude was examined with two-factor repeated measure ANOVA (Window (early, late) *X* folds (1–5)). There was an interaction effect (*F*_4,84_ = 3.657, *P* = 0.009, partial η^2^ = 0.148), confirming the impression that effect of *N*-folds on SPN amplitude changed between the early to late windows.

In the early window (350–450 ms), where SPN and *W* were closely related, there was a main effect of folds (*F*_4, 84_ = 5.541, *P* = 0.001, η^2^ = 0.209). There was a significant increase in SPN amplitude from 1- and 2-fold conditions (*t*(21) = 2.830, *P* = 0.010), a marginal *reduction* in SPN amplitude between 2- and 3-fold (*t*(21) = −2.012, *P* = 0.057), and then a significant *increase* from 3- to 4-fold (*t*(21) = 3.933, *P* = 0.001). Most variance at this early time window was captured the complex quartic contrast (*F*_1,21_ = 12.343, *P *= 0.002, η^2^ = 0.370, Fig. 12*D*).

In the late window (600–1000 ms), where SPN and *T* were closely related, there was again a main effect of folds (*F*_2.693, 56.563_ = 5.707, *P *= 0.002, η^2^ = 0.214). Here, the only significant increase in SPN amplitude occurred between 1- and 2-fold conditions (*t*(21) = 3.568, *P *= 0.002). Most variance in this later window was explained by the simple linear contrast (*F*_1,21_ = 12.573, *P *= 0.002, η^2^ = 0.374, Fig. 12*E*).

In the early window, *W* explained most variance in grand-average SPN amplitude (*R*^2^ = 0.952), while *T* was less successful (*R*^2^ = 0.592). This can be seen in the radar plot in Figure 12*D*, and also in the line graph, which showcases the characteristic dip in the 3- and 5-fold conditions (uniquely predicted by the holographic model). Linear mixed effects analysis confirmed *W* had a significant effect on SPN (SPN μV −4.115 W, χ^2^(1) = 9.817, *P *= 0.002). *T* also explained some variance SPN amplitude in the early window (SPN μV = 2.874 T, χ^2^(1) = 6.165, *P *= 0.013); however, *W* explained more variance in SPN amplitude than *T* ( χ^2^(1) = 10.147, *P *= 0.001).

In the later 600–1000 ms window (Fig. 12*E*), *T* explained more variance in grand-average SPN than *W* (*R*^2^ = 0.933 vs. 0.740). Although both *T* and *W* were significant predictors of SPN amplitude (SPN μV = −3.893 T, χ^2^(1) = 11.540, *P*  < 0.001; SPN μV = −3.913 W, χ^2^(1) = 8.706, *P* = 0.003), *T* explained more variance than *W* (χ^2^(1) = 5.364, *P* = 0.021).

Finally, we analyzed the individual *W* versus SPN and *T* versus SPN correlations in the early and late widows. There was a significant Window×Model interaction (*F*_1, 21_ = 22.924, *P* < 0.001, partial η^2^ = 0.522), because *W* correlations were significantly higher in the early window (*t*(21) = −2.601, *P* = 0.017), and *T* correlations were numerically larger in the late window (*t*(21) = 1.701, *P* = 0.104).

In summary, several kinds of analysis confirmed that *W* is a better fit in the early window, and *T* in the later window. Global Field Power this mirrored the SPN data closely, especially in the early window (Supplementary Material 4).

## Study 3: Discussion

The *N*-fold experiments re-affirmed some of the lessons from Study 1. Again, we see that SPN is closely related to *W*, but the relationship is stronger in an early period. The later SPN was more closely related to *T*. The results of the behavioral experiment were also better predicted by *T*. However, the behavioral data could be shaped by a ceiling effect, which gave near perfect performance whenever *N*-folds >1. This would result in data that happen to resemble the transformational predictions more closely, but for trivial reasons.

We also note that the predictions of the transformational model are not supported in other experiments. The transformational model cannot account for the difference between reflection and repetition, both of which involve invariance under rigid transformation, or the graceful degradation with noise (Palumbo et al. 2015). Overall, the holographic model predicts SPN amplitude much better than the transformational model.

## Study 4: Symmetry and Anti-symmetry

So far we have always produced symmetry by luminance matching. For example, black dots were paired with black or white were paired with white. The correlation between luminance in equivalent positions on either side of the axis was +1. However, it is also possible to produce patterns with “anti-symmetry,” where white dots are paired with black and vice versa (so the correlation is −1). The *amount of information* in symmetry and anti-symmetry patterns is identical. Does this mean symmetry and anti-symmetry are perceptually identical, and have the same *W*-load? Perhaps: but the holographic model is agnostic here—it assumes that matched elements are totally identical. Study 4 tested the scope of the holographic model by examining the SPN for anti-symmetry.

Study 4 had had a mixed design. There was one within-subjects factor (symmetry, anti-symmetry) and one between-subjects factor (1-fold, 4-fold). We expected that 4-fold symmetry would produce a larger SPN than 1-fold symmetry. However, we did not have an a priori prediction for anti-symmetry ERPs.

Tyler and Hardage (1996) proposed that symmetry and anti-symmetry are perceptually equivalent because all symmetry analysis feeds on “second-order” channels with large receptive fields that are insensitive to contrast polarity. This account predicts exactly the same SPN for symmetry and anti-symmetry. However, other studies have found that some kinds of symmetry and anti-symmetry are perceptually different (van der Helm and Treder, 2009). In fact, Mancini et al. (2005) concluded that symmetry perception feeds on luminance sensitive “first-order” channels with small receptive fields, while anti-symmetry is only discriminated by effortfully noting positional matches across the midline. This account suggests that anti-symmetry may not generate an SPN at all.

## Study 4: Stimuli

**Figure 13.**
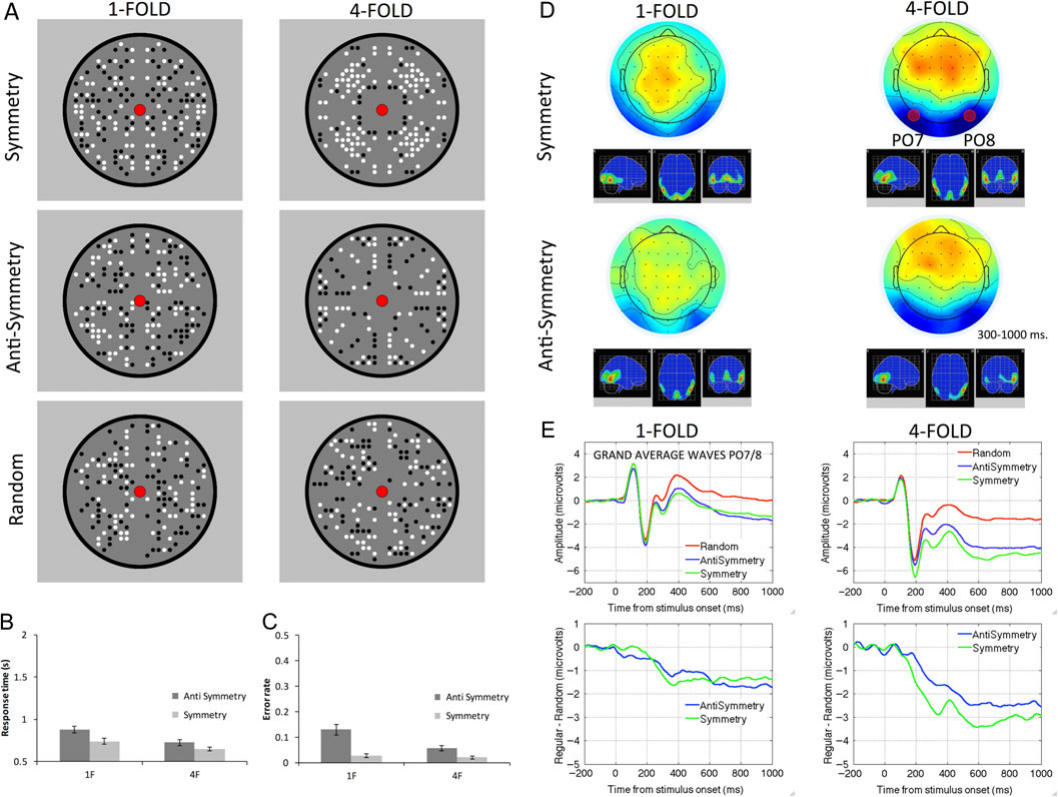
Study 4 stimuli and results. (*A*) Example symmetry, anti-symmetry, and random stimuli. Response times (*B*) and error rates (*C*) from the behavioral experiments. (*D*) Topographic difference maps and estimate of cortical sources in the 300–1000 ms window. (*E*) Grand-average ERPs and difference waves at PO7/8.

Example stimuli are shown in Figure 13*A*. These were generated with the same basic code as the *N*-folds experiment, with some modifications. A new invisible boundary was added to prevent the elements touching the perimeter and axes. Each cell in the grid had 40% occupation probability, and the dot was equally likely to be black or white. The symmetry and anti-symmetry patterns were identical in terms of positional information.

These parameters were chosen in light of the known effects of density on anti-symmetry perception (e.g. Wenderoth 1996, Mancini et al. 2005). By the standards of these previous studies, our arrays were relatively sparse, (~8.9 elements per deg^2^). At this density, anti-symmetry should be readily distinguishable from random, but not at the level where it was perfectly equivalent to symmetry.

As in Study 3, the pattern generation algorithm introduced a relationship between *N* dot variability, regularity and *N*-folds. The 1-fold had an average of 172 dots, and an SD of 15. The random patterns from the 1-fold experiment again had 172 dots on average, but the SD was 10. The 4-fold patterns had an average of around 157 dots, with an SD of 29 dots. The random trials in this experiment had the same mean, but an SD of only 10. We also note that 4-fold anti-symmetry patterns also had 90° luminance-matched rotational symmetry. Even if participants interpreted this as a rotation rather than anti-symmetry on a fraction of the trials, SPN amplitude may decrease. However, there was no luminance-matched rotation in the 1-fold patterns.

In each experiment, there were 288 trials in total; 144 were random, 72 were symmetrical, and 72 were anti-symmetrical.

## Study 4: Behavioral Experiments

Separate groups of 22 participants were involved in each behavioral experiment (1-fold, age 16–45, 8 male, 1 left-handed; 4-fold, age 16–54, 9 male, 2 left-handed). Participants discriminated regular (symmetry or anti-symmetry) from random patterns as quickly and accurately as possible.

RT results are shown in Figure 13*B*. There was a main effect of the between-subjects factor Folds (*F*_1,42_ =6.948, *P* = 0.012, partial η^2^ = 0.142), and the within-participants factor Regularity type (*F*_1,42_ = 103.916, *P* < 0.001, partial η^2^ = 0.712). There was also a Regularity type×Folds interaction (*F*_1,42_ =8.787, *P* = 0.005, partial η^2^ = 0.173) because the advantage for symmetry over anti-symmetry was greater in the 1-fold group (*t*(21) = 10.059, *P* < 0.001), although still present in than the 4-fold group (*t*(21) = 4.779, *P* < 0.001).

Error rate results were comparable (Fig. 13*C*). There was a main effect of Folds (*F*_1,42_ = 8.086, *P* = 0.007 partial η^2^ = 0.161) and Regularity type (*F*_1, 42_ = 56.602, *P* < 0.001, partial η^2^ = 0.574) and an interaction (*F*_1,42_ = 12.391, *P* = 0.001, partial η^2^ = 0.228), because the the advantage for symmetry was greater in the 1-fold group (*t*(21) = 6.149, *P* < 0.001) than in the 4-fold group (*t*(21) = 4.550, *P* < 0.001).

## Study 4: ERP Experiments

Separate groups of 22 participants were involved in each EEG experiment (1-fold, 16–54, 9 male, 2 left-handed; 4-fold, 18–36, 8 male, 3 left-handed, see Supplementary Material 5 for details).

SPN results are shown in Figure 13*D* and *E*. As expected, the SPN was larger in the 4-fold group than in the 1-fold group. The SPN was comparable for symmetry and anti-symmetry, but amplitude was slightly reduced for anti-symmetry in the 4-fold group. All conditions produced a comparable topographic difference map (Supplementary Material 5), and the SPN was always localized to extrastriate visual regions. There was no consistent right lateralization.

All symmetry and anti-symmetry patterns generated an SPN (regular–random <0, *t*(21) = 4.088, *P* = 0.001). Mixed ANOVA revealed that the SPN difference between 1- and 4-fold groups was significant (*F*_1,42_ = 9.579, *P* = 0.003, partial η^2^ =0.186). The difference between symmetry and anti-symmetry approached significance (*F*_1,42_ = 3.262, *P* = 0.078) as did the Regularity×Folds interaction (*F*_1,42_ = 3.537, *P* = 0.067). In the 4-fold group, symmetry produced a larger SPN than anti-symmetry (*t*(21) = 3.332, *P* = 0.003). However, there was no difference between symmetry and anti-symmetry in the 1-fold group, (*t*(21) = −0.043, *P* = 0.966).

However, the apparent similarity between 1-fold symmetry and anti-symmetry requires critical examination. The topographic maps from the 1-fold experiment certainly suggest a more substantial posterior negativity for symmetry (just not at electrodes PO7/8, Fig. 13*D*). Indeed, GFP was significantly higher for symmetry than anti-symmetry here (*t*(21) = 3.194, *P* = 0.004, see Supplementary Material 5 for full analysis).

## Study 4: Discussion

We found that the SPN-generating mechanisms in the extrastriate visual cortex responded to anti-symmetry both 1) without delay and 2) with only a small reduction amplitude. Furthermore, the effect of folds on SPN amplitude was independent of whether the display involved symmetry or anti-symmetry.

The SPN was very similar for 1-fold symmetry and 1-fold anti-symmetry at PO7/8 electrodes. However, GFP was lower for 1-fold anti-symmetry. Furthermore, two new experiments from our laboratory (not yet published) have also found a smaller SPN for 1-fold anti-symmetry. Participants were slower and less accurate to detect anti-symmetry in the 1-fold experiment. This again confirms that perceptual goodness of our anti-symmetry was reduced (although not by much, compared with repetition, for example).

Our results are not consistent with Tyler and Hardage (1996), who claimed symmetry and anti-symmetry are equivalent, or with Mancini et al. (2005) who predict a totally different brain response to anti-symmetry. The results of our Study 4 also go beyond the formalizations of the *representational* holographic model. We cannot assume that two patterns have the same *W* value unless elements are matched in every way. New *process models* are thus required to understand the slightly weaker SPN for anti-symmetry.

To this end, we propose that symmetry analysis in the extrastriate visual cortex can flexibly feed on either first-order (luminance sensitive) channels or on second-order (contrast sensitive) channels. First- and second-order channels can be modelled as distinct spatial frequency filters: *W*-load reflects *post-filter* representational strength, and the SPN indexes a *post-filter* neural response to symmetry. Perhaps the SPN amplitude *difference* for symmetry and anti-symmetry is inherited from below, that is, from the relative excitation of first- and second-order channels. Meanwhile, the extrastriate network may simply code positional correspondences in its input. The SPN is probably generated by the extrastriate network, hence the basic similarities in latency and topography for symmetry and anti-symmetry.

## Comparing Different Measures of Perceptual Goodness

In several experiments, there was an early peak where SPN data were most closely related to the holographic model. The SPN was right lateralized in Studies 1 and 2.

**Figure 14.**
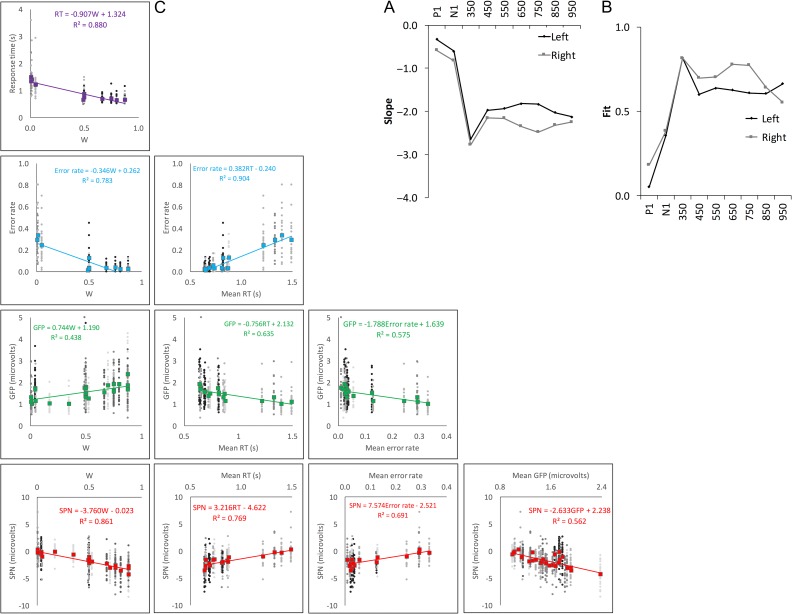
Multi-experiment results. *W* versus grand-average SPN slope (*A*) and fit (*B*) estimates in successive time windows from left and right posterior electrodes. (*C*) Scatter plot matrix showing agreement between five measures of perceptual goodness. SPN and GFP data are taken from the peak 300–400 ms window. Gray datapoints are individual participants; grayscales correspond to experiments with different samples. Grand-averages are shown in color. The trendline and regression equations correspond to colored grand-average data. *R*^2^ indicates the amount variation in grand-averages explained by the variable on the *x-*axis.

To capture these effects, Figure 14 shows multi-experiment estimates of slope (*A*) and fit (*B*) in each hemisphere, in successive time windows (based on grand-average data only, as in Fig. 3*A*). There is a peak at around 300–400 ms. We refer to the 300–400 ms window as the early SPN, which may index the goodness of initial perceptual representations (not secondary cognitive processes). Interestingly, right lateralization begins *after* the early SPN peak (see also sequential topographies in Supplementary Materials 2–5).

In each of our four new studies reported here, we have five different measures of perceptual goodness: A theoretical measure (*W*), two neural measures (early SPN and GFP) and two behavioral measures (response time and error rate). It is worth briefly examining consistency between these disparate measures by pooling data across all available experiments. Figure 14*C* shows the matrix of scatterplots with individual subjects and grand-averages overlaid. Linear mixed effects analysis found that every relationship was significant (*P* < 0.001).

Finally, we tested whether *W* explains more variance in the early SPN amplitude than the *E* and *N* terms individually (across experiments where this could be estimated). Indeed, we often set *W* by varying *E* while keeping *N* constant, and consequently *E* was positively correlated with *W* (*r* = 0.766). We found that *E* explained considerable variance in SPN amplitude (SPN = −0.011E, χ^2^ (1) = 151.450, *P* < 0.001), but not as much as *W* (SPN = −3.915 W, χ^2^(1) = 194.220, *P* < 0.001). Importantly, *W* explained significantly more SPN variance than *E* (χ^2^(1) = 56.310, *P* < 0.001). Similar analysis found that *W* also explained more variance than *E* in RT (χ^2^(1) = 232.660, *P* < 0.001) and Error rate (χ^2^(1) = 270.400, *P* < 0.001). Conversely, more GFP variance was explained by *E* than *W* (χ^2^(1) = 37.616, *P* < 0.001). Along with other analysis, this confirms that GFP is less precisely tuned to the predictions of the holographic model than the SPN itself.

## General Discussion

Visual inputs are highly predictable across space. It has long been recognized that the brain avoids wastefully coding all the redundant information. Instead, redundancy is compressed, and patterns that allow efficient compression are subjectively salient and quickly detected. For example, Attneave (1954) said that “*the good gestalt is a figure with some high degree of internal redundancy”* (p. 186). The holographic approach concurs, but provides a more detailed conceptualization of redundancy.

There are 20 regularities that have the “holographic property,” including reflection, repetition, rotation, and Glass patterns (van der Helm and Leeuwenberg 1996). These regularities can be divided up into substructures, and the resulting parts have the same regularity as each other. But this only holds if the patterns are partitioned into holographic identities. For reflection, each pair of points constitutes a holographic identity, for repetition, each pair of blocks is a holographic identity. In the holographic model, perceptual goodness of a pattern (*W*) is the number of holographic identities (*E*) divided by the total number of elements (*N*). Perceptual goodness is thus the ratio of amount of information compressed by the coding system (*E*) and total information in the patterns (*N*).

Combined analysis across all recordings found that SPN = −3.234 W, and *W* explained 86% variance in grand-average SPN amplitude. Similar slope and fit metrics were found in the individual studies. More fine-grained analysis showed that *W* was most closely related to the SPN in an early part of the SPN window. We did not predict this a priori, but it makes sense nevertheless. The holographic model was designed to explain the salience of initial perceptual representations, as opposed to subsequent cognitive processing or deployment of spatial attention. Therefore, one can see the early SPN as a *pure* measure of perceptual goodness. Conversely, psychophysical measures such as response time and error rate cannot assess perceptual goodness directly, without incorporating other factors that affect processing at secondary stages.

It is interesting that the SPN slope estimates were consistent across five analyses (Table 1), while other goodness measures were more sensitive to *W* range. This suggests that the SPN is relatively free from secondary influences, such as floor and ceiling effects, which can contaminate RT and error rate data. It also means we can confidently extrapolate from the results of a single study where *W* range is limited.

The holographic model is abstract: Nobody claims that the brain works out perceptual goodness in such a methodical and formulaic way. The visual system certainly never converts stimuli into symbol strings. Some see this silence about processing as a limitation (Wagemans 1999), while others argue that understanding the underlying structure of symmetry representations is an important pre-requisite (van der Helm and Leeuwenberg 1999). There has been a debate about the scope of the holographic model, and the nature and plausibility of its assumptions (Olivers et al. 2004; van der Helm and Leeuwenberg 2004). Without taking a strong position on all aspects of this debate, we note that perceptual research has often progressed by using abstract models than are not biologically plausible. For example, Hochberg and McAlister (1953) demonstrated that vision imposes the *simplest* interpretation on ambiguous Kopfermann cubes—where *simplicity* may be quantified as the number of line segments or angles. This was an important insight, even though the visual system surely never tabulates simplicity scores for each possible interpretation. Likewise, contemporary researchers have built abstract computational theories of perception using Bayesian methods—even without knowing how the brain codes probability (Knill and Pouget 2004). Abstract accounts can provide insight into brain function without considering neural implementation.

However, we have shown that the holographic approach captures *something* about the neural basis of perceptual goodness. *W*-load predicts the SPN amplitude very successfully. We can briefly comment on the possible neural implementation of *W*. At a neural level, *N* approximately equals the number of excited V1 channels. *E* approximately equals the number of spatial correspondences coded in extrastriate areas (e.g. the LOC). Other research has shown that V1 activation is often reduced when pattern elements can be perceptually organized into a unified gestalt (de-Wit et al. 2012). This gives a neural explanation for why *E* (i.e. spatial correspondence) and *N* (i.e. coding of individual elements) should be inversely related.

We could also increase biological plausibility by considering structure in V1-filter outputs rather than structure in dot pattern stimuli. By applying spatial filters any symmetrical image can extract aligned blobs, and blob alignment indicates the presence of reflectional symmetry (Dakin and Watt 1994). Further filter rectifications allow extraction of anti-symmetry in sparse displays (Mancini et al. 2005). There is no such blob alignment for repetition or rotation. This perhaps provides clues about the holographic distinction between point structures (for reflection) and block structures (for repetition and rotation). These links could be explored in future.

However, we caution that perceptual goodness is NOT a straightforward property of 2D natural images. Makin et al. (2015) found that when participants were attending to regularity, the SPN amplitude was related to *W of the object*, independent of view angle. The SPN was nearly identical for both 2-fold reflections presented in the frontoparallel plane and 2-fold reflections presented with perspective slant that degraded regularity in the image. Various perceptual operations, like perspective normalization and figure-ground segmentation, must stand between the front-end spatial filters and the upstream gestalt representations in the extrastriate cortex. The holographic model is primarily about the goodness of post-filter representations.

Why is it adaptive for the visual system to be tuned to certain holographic regularities? A fundamental purpose of the visual brain is to find non-accidental relationships. Sensitivity to certain holographic regularities may be useful because these regularities are commonly found in real objects (which are often the results of structure-preserving growth, van der Helm 2011). However, we do NOT think many SPN results can be explained by the biological relevance of faces or bodies. There are too many exceptions. To give just one example, the SPN in Study 2 was equal for 1-fold reflection (very face-like) and concentric Glass patterns (not at all face-like).

## Conclusions

The *W* metric from the holographic model predicts subjective perceptual goodness and psychophysical results. Here, we show that *W* also predicts the amplitude of the neural response to regularity. This is a major step forward in characterizing mid-level vision, where the conscious experience of structure and shape emerges.

## Supplementary Material

Supplementary material can be found at: http://www.cercor.oxfordjournals.org/

Supplementary Data
