# Supplementary material for: An Electrophysiological Index of Perceptual Goodness
Source: Cereb Cortex. 2016 Dec 26;26(12):4416–34. doi: 10.1093/cercor/bhw255 (PMC5193141; doi:10.1093/cercor/bhw255)
Supplement: Supplementary Data [file supp_26_12_4416__index.html]

An Electrophysiological Index of Perceptual Goodness — An Electrophysiological Index of Perceptual Goodness — Supplementary Data 

# An Electrophysiological Index of Perceptual Goodness

## Supplementary Data

Supplementary Data

- Supplementary Data - pdf file
- Supplementary Data - pdf file
- Supplementary Data - pdf file
- Supplementary Data - pdf file
- Supplementary Data - pdf file
