# Supplementary material for: An Electrophysiological Index of Perceptual Goodness
Source: Cereb Cortex. 2016 Dec 26;26(12):4416–34. doi: 10.1093/cercor/bhw255 (PMC5193141; doi:10.1093/cercor/bhw255)
Supplement: Supplementary Data [file supp_bhw255_Supplementary_Materials1MethodandWcalculations.pdf]

Supplementary Materials 1: Method and W calculations

EEG Methods

Here we outline some general features of the EEG methods used in all our studies. Participants had normal or corrected to normal vision, and were rewarded with either course credit or a small reimbursement for travel expenses. The studies had local ethics committee approval and were conducted in accordance with the declaration of Helsinki (revised 2008).

In all experiments participants sat 140 cm from a 60 Hz CRT monitor. EEG data was recorded from 64 scalp electrodes at 512 Hz using the BioSemi Active-Two system, in an electrically shielded and darkened room. Common Mode Sense (CMS) and Driven Right Leg (DRL) electrodes served as reference and ground. All experiments were programmed using Python and the open source PsychoPy libraries (Peirce, 2007). Patterns were generated afresh on each trial, so no participant ever saw the same pattern twice.

Participants were always asked to fixate centrally throughout the baseline and stimulus intervals. They then entered a response using their left or right index fingers, after the stimulus disappeared. No motor response was entered during the presentation period.

EEG Data was analysed using the EEGLAB toolbox for Matlab (Delorme and Makeig, 2004). Raw EEG data re-referenced to a scalp average, low-pass filtered at 25Hz, and downsampled to 128Hz. The data was epoched into -1 to 1.5 second epochs, with a -200 ms to 0 baseline. Blinks, eye movement and other gross artefacts were removed with independent components analysis (ICA). After ICA, epochs were rejected if amplitude exceeded +/- 100 μV. Grand Average ERPs were computed for all electrodes and conditions.

Most analysis focused on ERPs in posterior PO7/PO8 electrodes. The SPN was defined as difference between regular and random waves from 300-1000 milliseconds.

Statistical assumptions

In this article we report many ANOVAs, examining RT, error rate, SPN and GFP data. We applied the Greenhouse Geisser correction factor whenever the assumption of Sphericity was violated, and report adjusted degrees of freedom.

ANOVA also assumes that the samples are normally distributed. This assumption was often violated in our 22 participant samples. The Shapiro-Wilk test found that 14/20 (70%) of our RT samples deviated from normality. For Error rate, 16/20 samples deviated from normality (80%). For GFP, this was 10/23 (43%). The ERPs were typically normally distributed, with only 4/33 (12%) deviations (we have found that the SPN is typically normality distributed before, e.g. Makin et al., 2013).

One option would be to employ outlier removal or data transformation procedures before running parametric analysis. However, this involves many arbitrary and post hoc decisions, and does not always produce normally distributed data anyway. Instead, we double-checked all ANOVAs and t tests with equivalent non parametric tests (which do not require normally distributed samples). Of the 115 significant main effects and interactions reported in this paper, 101 could be replicated with non-parametric tests ($p < 0.05$). This replication rate of 87.8% rises to 94.8% if we set the significance threshold at 0.1, because there were several borderline significant non-parametric replications (0.1 is arguably justifiable, given that we usually made one-tailed a-priori predictions). This analysis confirms that our results were not driven by a few outlying data points.

Unsurprisingly, the normalized data used in statistical topography analysis was nearly all normally distributed (1187/1296 variables, 91.5%), so we did not back this up with non-parametric tests.

Finally, we note that our figures show individual participant data points as well as means. This makes it easy to visualize our sample distribution properties. We also illustrate metrics from individual subjects. This generally confirms the samples were quite homogenous: most participants behaved like the average participant.

Stimuli and W-load calculation

W loads were computed according to the rules in van der Helm and Leeuwenberg (1996), and also described elsewhere (van der Helm, 2010). This section explains the W-load concept, as well as the stimuli used in these experiments. W = E/N, where E is evidence for regularity, and N is total information: So, the amount of regularity (somehow calculated) is coded as a proportion of the total information in the patterns (somehow calculated).

It is instructive to first consider the W-loads for multiple reflectional symmetries. If there is just 1 axis of reflection, E is the number of pairs, and N is the number of elements, so E/N = 0.5. We can also see that half the pattern is new information, but the other half is redundant information. For 2-Fold reflection, we can find pairs across one axis; so half the pattern is again redundant. In the remaining half, there is another local axis of symmetry; so half of the second half is also redundant. In total only 0.25 of the pattern is informative, and 0.75 is redundant. The W-load is 0.75 for two-fold reflection. For 4-Fold symmetry, there is a further nested local reflection in the remaining quarter, only 0.125 of the pattern is informative, and the remaining 0.875 is redundant. W-load is 0.875 for 4-fold reflection. So far, W seems to be directly linked to redundancy. However, for 3-Fold symmetry, W =0.667,

even though 0.833 of the information is redundant. This is because the local regularities overlap, and only one of these can be exploited and coded by the formalization of the holographic model.

For repetition, W is E/N again, but E is the number of repeating blocks – 1, while N is again the number of elements. This is different from reflection, where E is the number of dot-pairs. Understanding this difference gets to the heart of the holographic approach to regularity. Holographic regularities can be divided into parts, and each of the resulting parts has the same kind of regularity as each other and as the original. For example, a reflectional symmetry with 100 dots can be chopped into 5 substructures with 20 dots each. The resulting substructures are all reflections, with the same regularity as each other, and with the same regularity as the original 100-dot pattern. The same is true if we divided the 100-dot pattern into substructures of other sizes as well. We thus say reflection possesses the '*holographic property*' (meaning that all substructures have the same regularity, which is also the regularity of the original). We can put this another way: A reflectional symmetry can be grown and extended by one dot pair at a time, to an infinite size, and the holographic property remains after the expansion. Because reflection can be extended one pair at a time, it is said to have a *point structure*. Repetition also has the holographic property, but only if we extend it by one repeating block at a time. For instance, a 10 repeat pattern can be chopped into two blocks of 5 repeats, and both sub-structures are repetitions. We can extend the pattern by another 10 repeats, divide it up into substructures, and all these have the same repetition structure. But we cannot add half a block and retain the holographic property. Repetition thus has a *block structure* according to the holographic model, because the minimum unit for growth is an entire block, while for reflection the minimum unit for growth is a single pair.

In the experiments reported below, we calculate the W-load of the stimuli based on the ideas of the holographic model. In doing so, we made various assumptions. These caveats are important. Nevertheless, we think that the stated W-loads are a fair representation of the ideas behind the holographic model, and any alternative calculations of W would not substantially alter the picture. Importantly, the rank ordering of W-loads is always preserved, even if there are minor ambiguities.

The patterns used by Makin et al. (2012) were made of solid shapes, not separate dots (Figure 1A). There were two axes of symmetry, so we give these a W-load of 0.75. However, the black elements overlapped and formed perceptual groups, so we cannot really say how many elements or pairs there were. This is not too problematic, because however one conceptualizes N in these patterns, it always has the same proportional relationship to E.

For the patterns used by Makin et al. (2013), there were always 22 dots, with 11 on each side of the midline (Figure 1B). The reflections had 1 vertical axis, E was 11, N was 22, so consequently the W-load was 0.5. For repetition, there were 2 repeated blocks, E = 2-1 = 1, N = 22, so W = 1/22 = 0.045. Rotation is treated in the same way as repetition by the holographic model, and W was 0.045 for rotation as well. However, the size and colour of the dots was also matched, and the dots could overlap slightly. This meant different perceptual groupings and interpretations were possible. We cannot say for sure that participants perceived 22 elements rather than a smaller number of larger groupings. The real W-load for repetition and rotation may be underestimated here, because we are overestimating N, the number of elements.

The behavioural work from Csathó, van der Vloed and Van der Helm (2003) is relevant here. They found that salient perceptual substructures within each half strengthen

repetition but weaken reflection. We ignored the perceptual effects of multi-element substructures when calculating W. This is not so much of a problem for Makin et al. (2012) because all the patterns were reflections. For Makin et al. (2013), all patterns were quite homogenous with regard to the number of such perceptual groupings, and the ordinal goodness ranking of reflection > repetition = rotation was certainly preserved.

There is also a question about elements that fall exactly on the axis of symmetry. These are ambiguous. They are clearly contributing to symmetry itself, but they are not pairs. The holographic model does not have a formula for coding the contribution of centrally positioned elements. Nevertheless, central elements were present in Makin et al., (2012), Palumbo et al., (2015) and in Study 3. We assume that W can be determined purely by the number of axis in these experiments, and do not make any adjustments to deal with the central elements.

Finally, there is a problem of residual regularity in the random patterns. Here the elements on either side of the axes were chosen independently. There was thus a small amount of *accidental* regularity in the random stimuli. The degree of accidental pairing varied between experiments, and it was higher in experiments with higher element density. W was always close to zero but also always positive for random patterns, with the actual value changing from one stimulus to another (unlike the fully regular patterns in which the value was constant).

The imperfect 4-fold symmetry in Palumbo et al. (2015) deserves special consideration in this regard. The stimuli had a number of symmetrically positioned dots, and a number of randomly positioned dots. The proportion of symmetrically positioned dots (pSymm) was set at 1, 0.8, 0.6, 0.4, 0.2 or 0. The W-load for the perfect 4-fold symmetry was 0.875. To calculate the W-load for imperfect symmetries, we simply multiplied 0.875 by

pSymm value, giving W-loads of 0.875, 0.7, 0.525, 0.35 and 0.175. With average density of 40%, independent random positioning on either side of an axis produces an average accidental dot-pairing rate of $0.4^2 = 0.16$. However, there were 4 folds, so the average accidental pairing rate for the random patterns was $0.4^2 + 0.4^3 + 0.4^4 + 0.4^5 = 0.26$. As pSymm increased, the accidental pairing rate reduced to 0.21, 0.16, 0.10, 0.05 and 0 (where all pairing was deliberate). These figures give the impression that the advertised pSymm values are a gross underestimate, and more so for the low pSymm patterns. However, the deliberate symmetry was 4-fold, the accident pairs were usually across just 1 axis, and very rarely more than two. Therefore, most of the regularity in this experiment was deliberate regularity, not accidental pairing. We think that it is reasonable to ignore accidental pairing when calculating W for these patterns.

Alternatively, it could be argued that advertised W-loads were overestimates, because random dots in one segment hinder coding of nested reflections in multi-axis patterns. Perhaps these two diverging complications cancel each other out. In any case, the W-loads in the pSymm experiment should be treated as approximations, but importantly, the approximations preserve the ordinal ranking of W-loads across the 5 conditions.

References

Csathó, Á., van der Vloed, G., & van der Helm, P. A. 2003. Blobs strengthen repetition but weaken symmetry. Vision Research, 43, 993–1007.

Delorme, A., and Makeig, S. 2004. EEGLAB: an open source toolbox for analysis of single-trial EEG dynamics including independent component analysis. Journal of Neuroscience Methods. 134: 9-21.

Makin, A. D. J., Rampone, G., Pecchinenda, A., and Bertamini, M. 2013. Electrophysiological responses to visuospatial regularity. Psychophysiology. 50: 1045-1056.

Makin, A. D. J., Wilton, M. M., Pecchinenda, A., and Bertamini, M. 2012. Symmetry perception and affective responses: A combined EEG/EMG study. Neuropsychologia. 50: 3250-3261.

Palumbo, L., Bertamini, M., & Makin, A. 2015. Scaling of the extrastriate neural response to symmetry. Vision Research, 117, 1–8.

Peirce, J. W. 2007. PsychoPy - Psychophysics software in Python. Journal of Neuroscience Methods, 162: 8-13.

van der Helm, P. A., and Leeuwenberg, E. L. J. 1996. Goodness of visual regularities: A nontransformational approach. Psychological Review. 103, 429-456.

van der Helm, P. A. 2010. Weber-Fechner behavior in symmetry perception? Attention Perception and Psychophysics. 72: 1854-1864.
