# Supplementary material for: An Electrophysiological Index of Perceptual Goodness
Source: Cereb Cortex. 2016 Dec 26;26(12):4416–34. doi: 10.1093/cercor/bhw255 (PMC5193141; doi:10.1093/cercor/bhw255)
Supplement: Supplementary Data [file supp_bhw255_Supplementary_Materials2.pdf]

Supplementary Materials 2: Extra information for study 1

EEG Method

In the EEG experiment, participants were required to fixate throughout both the 1.5 s baseline and 1.5 s stimulus presentation periods. Furthermore, they did not make a speeded response. Instead, they entered their judgment of Regular (i.e. either reflection or repetition) or Random *after the stimulus disappeared*. In half the trials the left key was used to report Regular and the right key was used to report Random. In the other half of the trials, this was response mapping was reversed (left key for Random; right key for Regular). This design meant that while the stimulus was on the screen the participant did not know which mapping would be presented. This design prevented lateralized anticipatory motor activity when the stimuli was present on the screen.

Error rates varied between conditions: Mean error rate was 11% on the random trials, 4% on the reflection trials and 21% on the repetition trials. In the repetition condition, error rate increased with N dots (from 15 to 24%). We did *not* exclude error trials from EEG analysis. This differs from behavioral RT data, where error trials were excluded.

EEG data was segmented in -1 to +1.5 second epochs, with a -200 to 0 ms baseline. ICA was used to remove gross artefacts from individual datasets. The average number of components removed was 9.36 (min = 3 max = 18). After ICA cleaning, trials where amplitude exceeded +/- 100 μV at any electrode were removed. Exclusion rate was 5.8%, and very similar across conditions (min 5.2%, max 6.4%). SPN was calculated as the difference between each condition and the average random wave, from 300 to 1000 ms, at PO7 and PO8 electrodes.

Global Field Power

Global Field Power (GFP) is the standard deviation of amplitude across the 64 electrodes at a particular time point. The more color-variation there is in a topographic map, the higher the GFP. GFP takes all the electrodes into account, and we can use this to check that results are not problematically dependent on electrode choice. Figure 6E shows regular – random different maps (300-1000 ms). In general, high W patterns produced topographies with higher GFP.

First GFP data was tested with 2 factor repeated measures ANOVA [2 (Regularity (Reflection, Repetition) X 3 (N Dots (20, 60, 100)]. As expected, GFP was higher in the reflection condition than the repetition condition (F (1,21) = 12.422, p = 0.002, partial η^2 = 0.372). There was no main effect of N Dots (F (2,42) = 0.245, p = 0.784). However, there was also no Regularity X N Dots interaction (F (2,42) = 2.643, p = 0.083). This is different to the SPN data, where this predicted interaction effect was found.

Next we ran regression analysis to examine the relationship between W and GFP. Results are shown in Supplementary Figure 2.1. Linear mixed effects analysis found that a significant effect of W on GFP (GFP (μV) = 0.614W, χ^2 (1) = 10.338, p = 0.001, Supplementary Figure 2.1A). W explained most variance in average GFP (R^2 = 0.884). The radar plot in Supplementary Figure 2.1B illustrates overlap between normalized W and GFP. The same basic pattern was found in 18/22 participants (mean R^2 = 0.35, with negative slopes coded as R^2 = 0 Supplementary Figure 2.1C and D). Like the SPN, the W vs. GFP correlation was stronger in the first half of the 300-1000 ms interval (Supplementary Figure 2.1E).

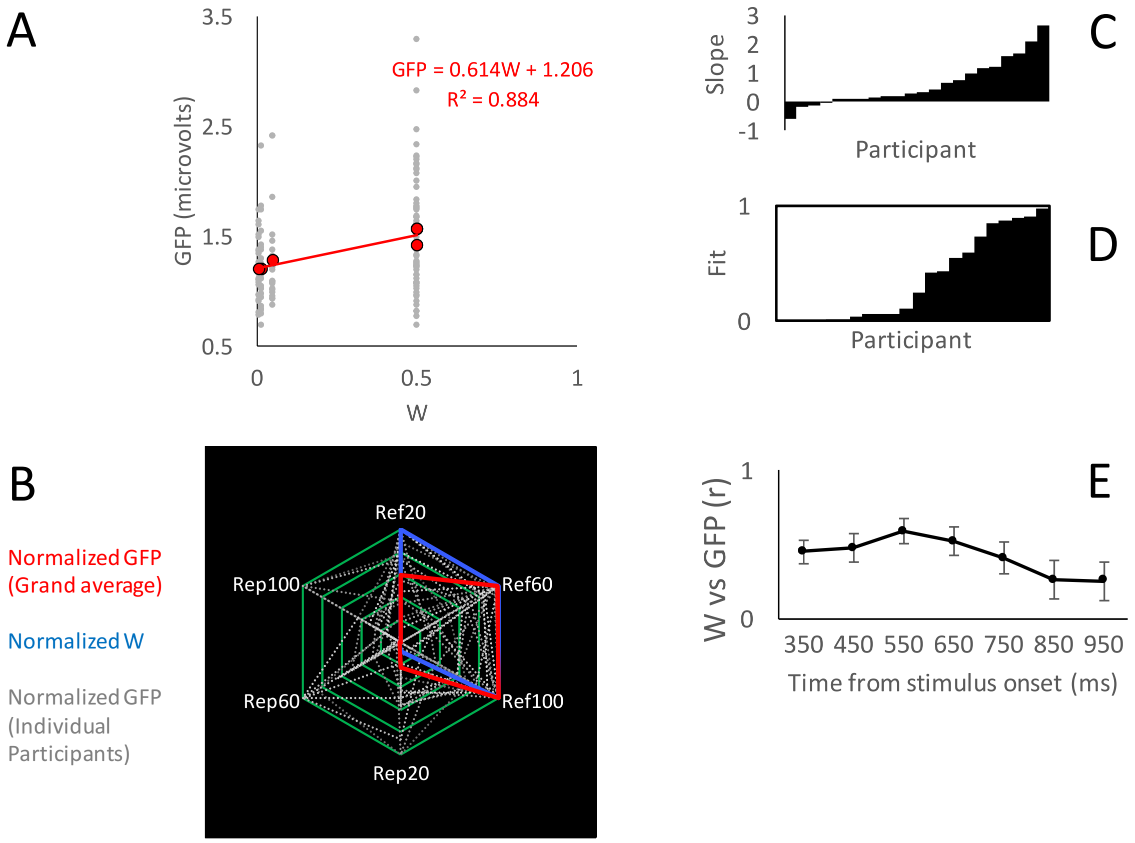

Supplementary Figure 2.1. Global Field Power analysis. A) Regression analysis of W vs. GFP relationship (Grand average in red, individual participants in grey, R^2 indicates the variance in *grand-average* SPN explained by W). **B)** Overlap between normalized GFP and W. **C)** Individual participant slope metrics. **D)** Individual fit metrics. **E)** Correlation between W and GFP in successive 100 ms. time bins through the SPN interval.

Statistical topography analysis

Amplitude and topography are inevitably interconnected. Any change in ERP topography will alter amplitude at each electrode, and conversely, change in amplitude at a subset of electrodes will alter topography. We put a lot of emphasis on SPN amplitude measured at PO7/8 electrodes, and tacitly assume that SPN topography was approximately the same in all conditions. But was our *assumption of topographic invariance* valid? Here we statistically analysed the topographies to address this concern.

A simplistic approach is to measure the correlation between amplitude at the 64 electrodes in pairs of grand-average topographic maps. After all, if two topographic maps were identical, the corresponding amplitudes would be perfectly positively correlated. We can see that inter-correlations were strong in the four conditions which produced a significant SPN (Supplementary Figure 2.2A). However, there is no principled way to judge whether these correlation coefficients are *high enough* to satisfy our assumption of topographic invariance.

We therefore ran a more detailed statistical topography analysis based on the approach of McCarthy and Wood (1985). More specifically, we obtained amplitude at each electrode and subtracted the data from the random condition, giving SPN for that participant and condition. We then normalized these 64-point vectors so that highest amplitude was 1 and lowest was 0. Specifically, for each of the 64 electrodes in the difference map, we 1) subtracted minimum amplitude at any electrode, and divided the result by the difference between maximum and minimum electrodes. This normalization is designed to remove amplitude differences that would otherwise bias topographic analysis (although see Luck, 2005 for critique of statistical topography analysis).

We then chose a 3X3 grid of scalp regions a-priori (shown in supplementary Figure 2.2B) and ran a 3 factor repeated measures ANOVA [2 Regularity (Reflection, Repetition) X 3 N Dots (20 60 100) X 9 Area (Front Left … Back right)] on the normalized data.

The normalized data used in this ANOVA is plotted in supplementary Figure 2.2C. The SPN is represented here as shorter columns in the back positions of the 3X3 column grid, particular on the right side.

A

	Ref 20	Ref 60	Ref 100	Rep 20	Rep 60	Rep 100
**Ref 20**						
**Ref 60**	0.85					
**Ref 100**	0.79	0.95				
**Rep 20**	0.70	0.63	0.54			
**Rep 60**	0.67	0.73	0.76	0.58		
**Rep 100**	0.01	0.20	0.31	0.06	0.27	

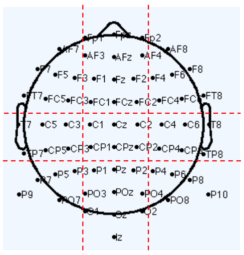

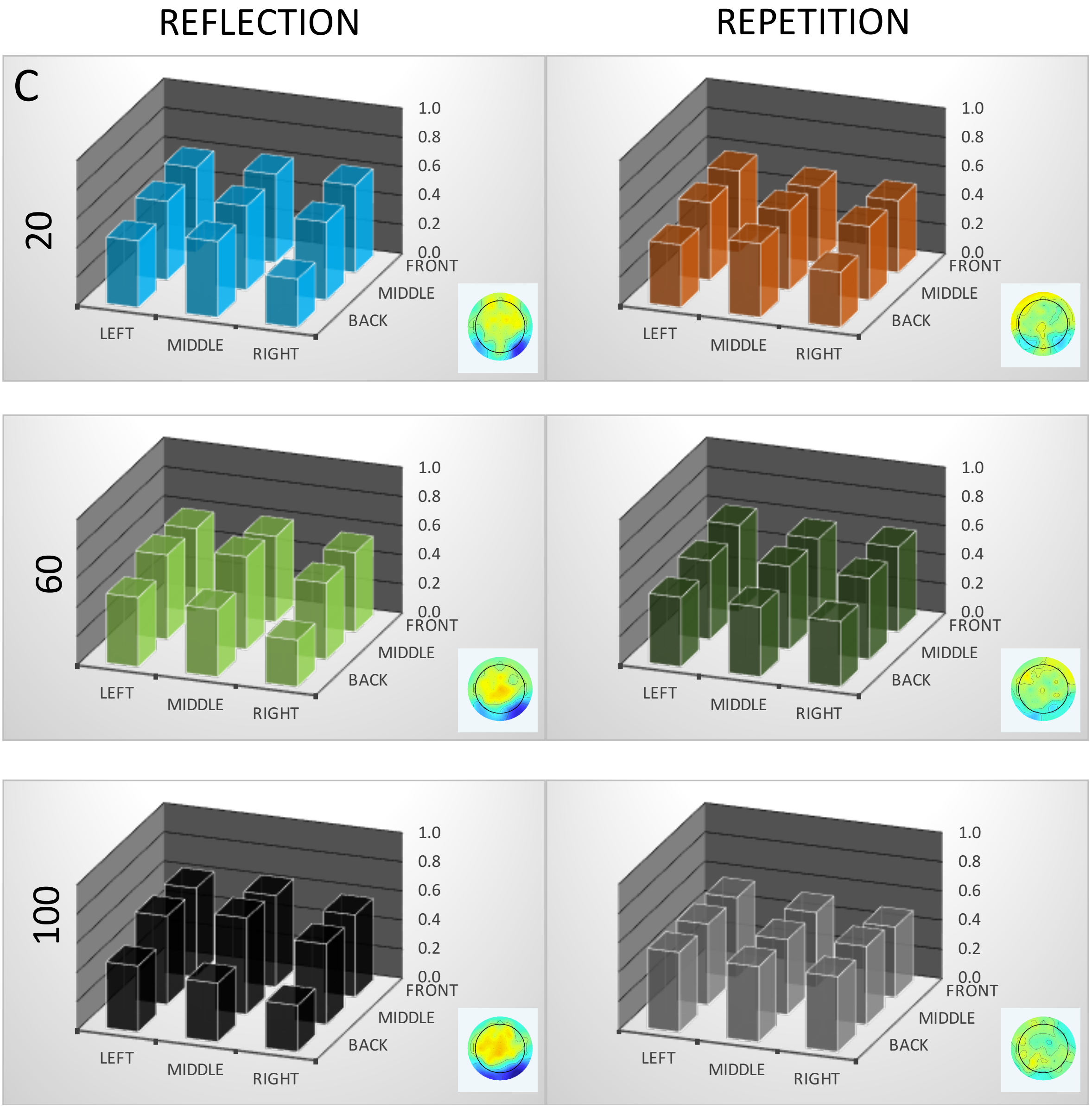

Supplementary Figure 2.2. Statistical topographic analysis of the 300-100 ms regular – random difference maps. **A)** Inter condition correlations between the 64 amplitudes at each electrode (+1 = identical topographies in two conditions). **B)** The nine electrode clusters used for statistical analysis. **C)** Grand average normalized amplitude at each cluster (inset shows the original topographic difference map for comparison). The SPN manifests as reduced amplitude at back columns, particularly on the right. Each panel shows one condition (Reflection, Repetition) X (20, 60 100 dots).

Crucially, if the assumption of topographic invariance is correct, then there should be no interactions involving the factor Area, even when there is a main effect of Area. However, given that there was no SPN in the Repetition 60 and Repetition 100 dot conditions, we expected interactions involving Area in our initial ANOVA, and indeed, there was a 3-way interaction between all factors (F (5.766, 121.092) = 2.763, $p = 0.016$, partial $\eta^2 = 0.116$), as well as a strong main effect to Area (F (3.186, 66.908) = 17.931, $p < 0.001$, partial $\eta^2 = 0.461$).

We next consider the reflection conditions independently. For reflection, we claim there is a similar SPN for 20, 60 and 100 dot conditions, and tacitly assumed that topography was fixed as well. As expected, analysis of the reflection conditions found that there was a main effect of Area (F (3.327, 69.865) = 21.451, $p < 0.001$, partial $\eta^2 = 0.505$), and importantly, no N Dots X Area interaction (F (6.082, 127.712) = 2.006, $p = 0.069$). From this, we could conclude that SPN topography was comparable in the 3 reflection conditions with differing numbers of dots.

However, this conclusion does involve theoretically interpreting a null result, which is notoriously problematic. The p value from an ANOVA tells us the probability of getting the observed data if the null hypothesis (H0) is true *(p Observed data| H0).* It does not tell us the probability of the null hypothesis being true give the observed data (*pH0 | Observed data*). However, it is possible to estimate the pH0|Oberseved data using Bayesian alternatives to null hypothesis testing, as described by Masson (2011). This gave a pH0 estimate for the N Dots X Area interaction was > 0.999, so we can be more confident in claim topographic similarity in the reflection conditions of study 1 (For comparison, the pH0 estimate for Area was just 0.069, and consequently pH1 was 0.931).

We next considered the Repetition 20 trials, where there was a significant SPN at the PO7/8 electrodes. Was topography for Repetition 20 similar to the SPN in the reflection conditions, as we assume? There was no Regularity X Area interaction when Repetition 20 was compared to Reflection 20 ($F (3.279, 68.867) = 1.504$, $p = 0.218$, $pH0 = 0.987$) or to Reflection 60 ($F (2.773, 58.242) = 1.678$, $p = 0.185$, $pH0 = 0.969$). However, there was a Regularity X area interaction when Repetition 20 was compared to Reflection 100 ($F (3.608, 75.774) = 3.636$, $p = 0.012$, partial $\eta^2 = 0.147$), showing that these two topographies were not identical.

Despite this exception, statistical topography analysis broadly confirms our assumption that SPN topography is invariant, while SPN amplitude varies according to the W-load of the patterns. Certainly the main effect of Area was much larger than any Area X Regularity interactions.

Evolution of the neural symmetry response across the SPN window

Next we then ran further analysis to quantify changes to SPN signal during the 300 to 1000 ms window. For this, we broke the 300-1000 ms SPN window in seven consecutive 100 ms sub-windows [(300 – 400), (400 – 500), (500 – 600), (600 – 700), (700 – 800), (800-900) and (900-1000 ms)].

Supplementary Figure 2.3 shows topographic difference maps (Regular – Random) in the seven 100 ms sub-windows. Although the topographies clearly change over time, there was always a substantial posterior negativity in the Reflection 20, 60 and 100 dot conditions, and a weaker SPN in the Repetition 20 dot conditions.

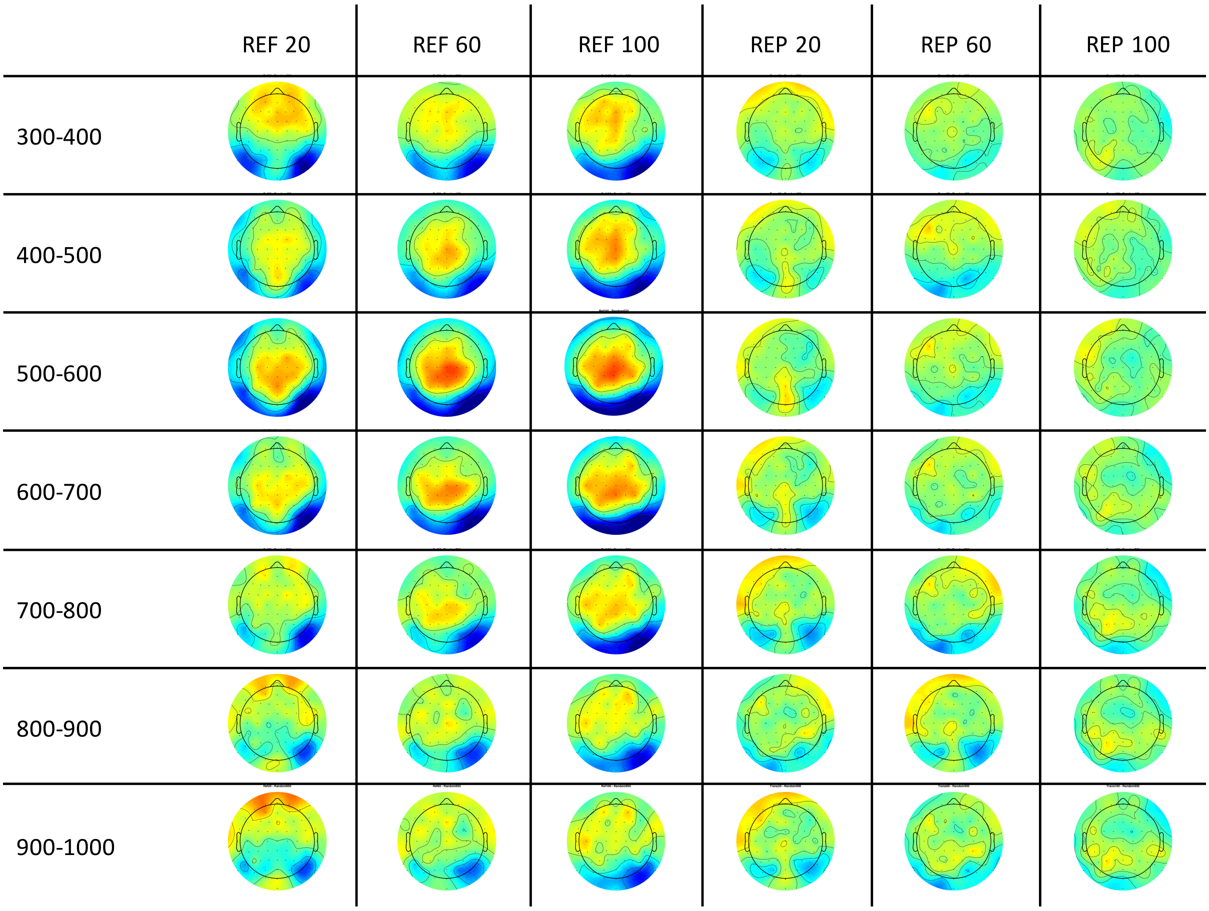

Supplementary Figure 2.3. Sequential topographies across the 300-1000 ms interval. Topographic maps regular-random differences in consecutive 100 ms time windows for the six conditions. Each column corresponds to a one condition; each row corresponds to a time window.

Statistical topography analysis suggested that the 3 reflection conditions were all the same at most time windows (Maximum interaction Regularity X area interaction, F (4.663, 97.922) = 1.947, p = 0.098, partial η^2 = 0.085, pH0 = 0.998), apart from 400-500 (F (5.432, 114.067) = 2.997, p = 0.012, partial η^2 = 0.125) and 500-600 ms (F (5.960, 125.159) = 2.254, p = 0.043, partial η^2 = 0.097), and these weak effects would not survive correction for multiple comparisons. In contrast, the effect of main effect of Area was present throughout (p < 0.001, partial η^2 > 0.259), although it declined at the later windows.

There were many cases where the topography of the Repetition 20 condition differed significantly from the reflection conditions in the early windows (8/9 significant Regularity X Area interactions), but fewer after 600 ms (1/12 significant Regularity X Area interactions). This analysis suggests the repetition 20 topography may become more like the reflection topography as we approach the end of the SPN window. Perhaps it takes longer for the extrastriate cortex to find repetition than reflection? However, this is a tentative suggestion, certainly the REP20 SPN wave at PO7/8 did NOT have a delayed onset (Figure 6D).

We next ran regression analysis on the relationship between W, Sustained Posterior Negativity (SPN) and Global Field Power (GFP) at each time point. The results are illustrated in Supplementary Figure 2.4, along with multi-level regression analysis. It can be seen that W was a significant predictor of both SPN and GFP at all time windows. However, W was better at predicting ERP patterns in the early part of the SPN interval.

To examine this temporal evolution statistically, we measured correlation coefficients from each participant, and analysed change over the 7 time bins with a 1-factor repeated measures ANOVA (Figure 6J). The W vs. SPN correlation became less negative across the interval for the SPN (F (2.466, 51.794) = 5.864, $p = 0.003$, $\eta^2 = 0.218$). This was characterized by a significant linear contrast (F (1,21) = 7.676, $p = 0.011$, $\eta^2 = 0.268$). However, we note that the mean correlation coefficient across the 22 participants was significantly less than zero at all time windows ($p < 0.010$).

300 – 400
SPN (µV) = -3.607W -0.229
χ^2 (1) = 18.494, $p < 0.001$
R^2 = 0.850

GFP (µV) = 0.676W + 1.066
χ^2 (1) = 13.201, $p < 0.001$
R^2 = 0.893

400 – 500
SPN (µV) = -2.466W -0.400
χ^2 (1) = 10.474, $p = 0.001$
R^2 = 0.777

GFP (µV) = 0.690W + 1.153
χ^2 (1) =10.398 $p = 0.001$
R^2 = 0.886

500 – 600
SPN (µV) = -3.879W -0.203
χ^2 (1) = 20.132, $p < 0.001$
R^2 = 0.940

GFP (µV) = 1.058W
χ^2 (1) = 11.850, $p = 0.001$
R^2 = 0.945

600 – 700
SPN (µV) = -3.662W-0.206
χ^2 (1) = 16.188, $p < 0.001$
R^2 = 0.835

GFP (µV) = 0.957W + 1.321
χ^2 (1) = 12.669, $p < 0.001$
R^2 = 0.850

700 – 800
SPN (µV) = -2.285W – 0.531
χ^2 (1) = 11.780, $p = 0.001$
R^2 = 0.639

GFP (µV) = 0.660W + 1.428
χ^2 (1) = 10.982, $p = 0.001$
R^2 = 0.891

800 – 900
SPN (µV) = -2.121W – 0.372
χ^2 (1) = 10.835, $p = 0.001$
R^2 = 0.627

GFP (µV) = 0.476W + 1.497
χ^2 (1) = 5.489, $p = 0.019$
R^2 = 0.796

900 – 1000
SPN (µV) = -1.995W – 0.334
χ^2 (1) =8.314, $p = 0.004$
R^2 = 0.671

GFP (µV) = 0.495W + 1.563
χ^2(1) = 6.726 p =0.010
R^2 = 0.825

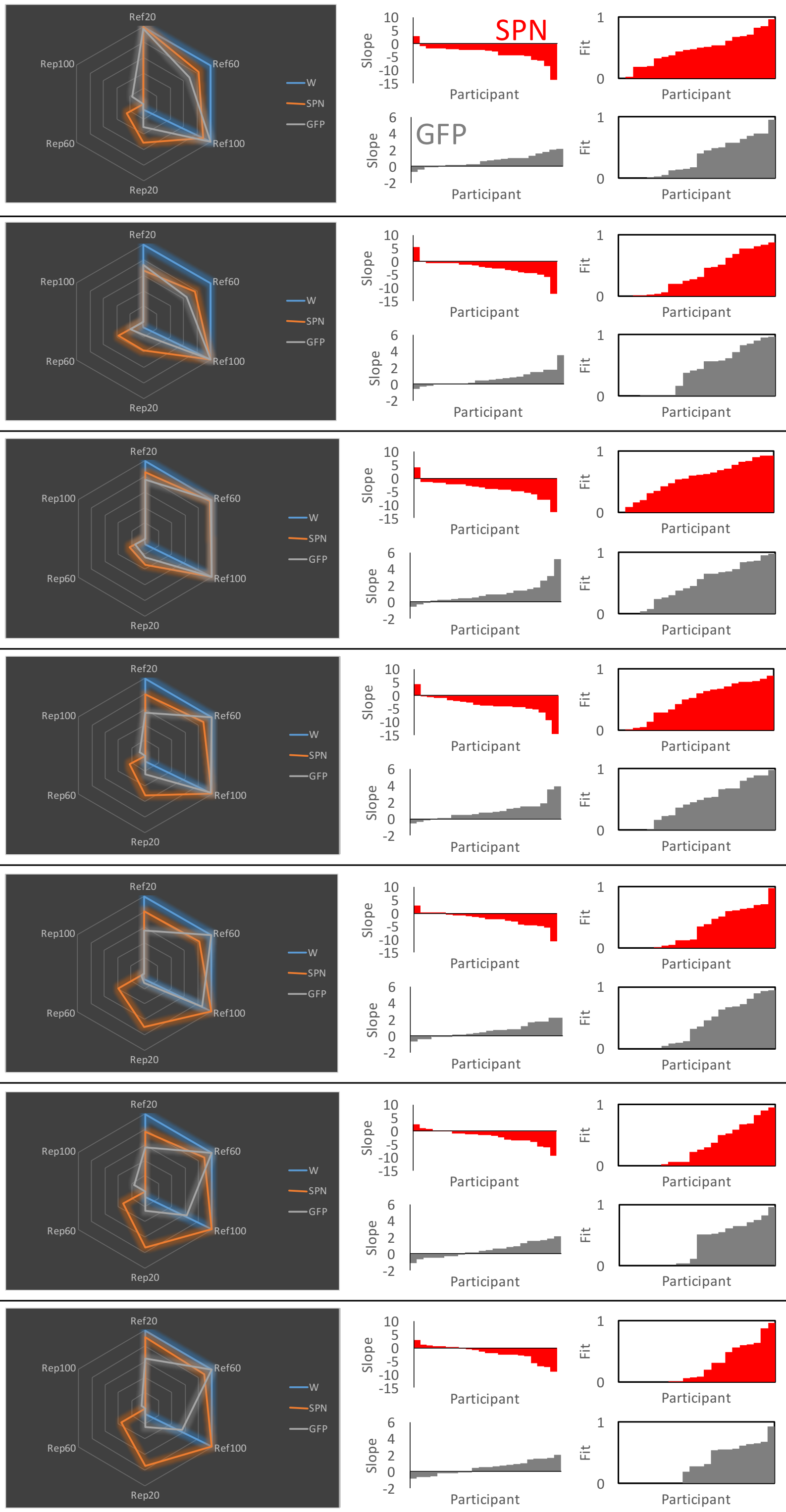

Supplementary Figure 2.4. W vs. SPN relationship across the 300-1000 ms interval. Each row shows various forms of regression analysis applied to ERP data in a 100 ms window. Details of the multilevel regression analysis are shown in the left. Radar plots show normalized W, grand average SPN and GFP overlaid. Right panels show equivalent slope and fit from each individual participant, organized cumulatively.

We also explored changes in the W vs. GFP correlation in the same way (Supplementary Figure 2.1E). The main effect of Time was only marginally significant ($F (2.393,50.250) = 2.488$, $p = 0.084$), and the first significant contrast was quadratic ($F (1,21) = 8.324$, $p = 0.009$, $\eta^2 = 0.284$). The mean correlation between W and GFP was significant in the first 5 time windows ($p < 0.002$) but only marginal in the last 2 ($p = 0.060$; $p = 0.064$).

In summary, W predicted the amplitude of the neural response to symmetry throughout the SPN window (300 to 1000 ms.) but W explains more SPN and GFP variance in the early part of the SPN interval.

References

Luck SJ. 2005. *An introduction to the event-related potential technique*. Cambridge, Mass: MIT press.

Masson MEJ. (2011). A tutorial on a practical Bayesian alternative to null-hypothesis significance testing. Behav Res Methods. *43*: 679–690.

McCarthy G, Wood CC. 1985. Scalp distributions of Event-Related Potentials - an ambiguity associated with Analysis of Variance models. *Electroencephalogr Clini Neurophysiol*, *62*:203–208.
