# Supplementary material for: An Electrophysiological Index of Perceptual Goodness
Source: Cereb Cortex. 2016 Dec 26;26(12):4416–34. doi: 10.1093/cercor/bhw255 (PMC5193141; doi:10.1093/cercor/bhw255)
Supplement: Supplementary Data [file supp_bhw255_Supplementary_Materials3.pdf]

Supplementary Materials 3: Extra information for study 2

EEG Method

As with study 1, participants were required to fixate throughout the 1.5 second baseline and 1.5 second stimulus presentation intervals on each trial. Judgments were reported after stimulus offset, with unpredictable response key mapping. Mean error rate was 13% for random, 9% for Glass, 3% for reflection, and 26% for repetition (comparable to error rate for repetition in study 1 EEG experiment). EEG data pre-processing was identical to study 1. On average 9.55 components were removed from each data set (min = 3, max = 22). Around 11% of trials were removed because amplitude exceeded +/- 100 μV at any electrode (min 10.5%, max 11.6%).

Global Field Power

Global Field Power (GFP) results are shown in Supplementary Figure 3.1. There was a main effect of Regularity on GFP ($F(2, 42) = 4.723$, $p = 0.014$, $\eta^2 = 0.184$). GFP for repetition was lower than for Glass ($t(21) = 2.908$, $p = 0.008$) or reflection ($t(21) = 2.787$, $p = 0.011$).

Supplementary Figure 3.1A illustrates the relationship between W and GFP. W was a significant predictor of GFP (GFP (μV) = 0.706W, $\chi^2(1) = 7.775$, $p = 0.005$). W explained nearly all variance in average GFP ($R^2 = 0.995$). This is highlighted in the radar plot in Supplementary Figure 3.1B. The same relationship was apparent in 16/22 participants (mean $R^2 = 0.406$, with negative slopes coded as $R^2 = 0$, Supplementary Figure 3.1C and D). The mean correlation between W and GFP declined slightly at the end of the interval (Supplementary Figure 3.1E).

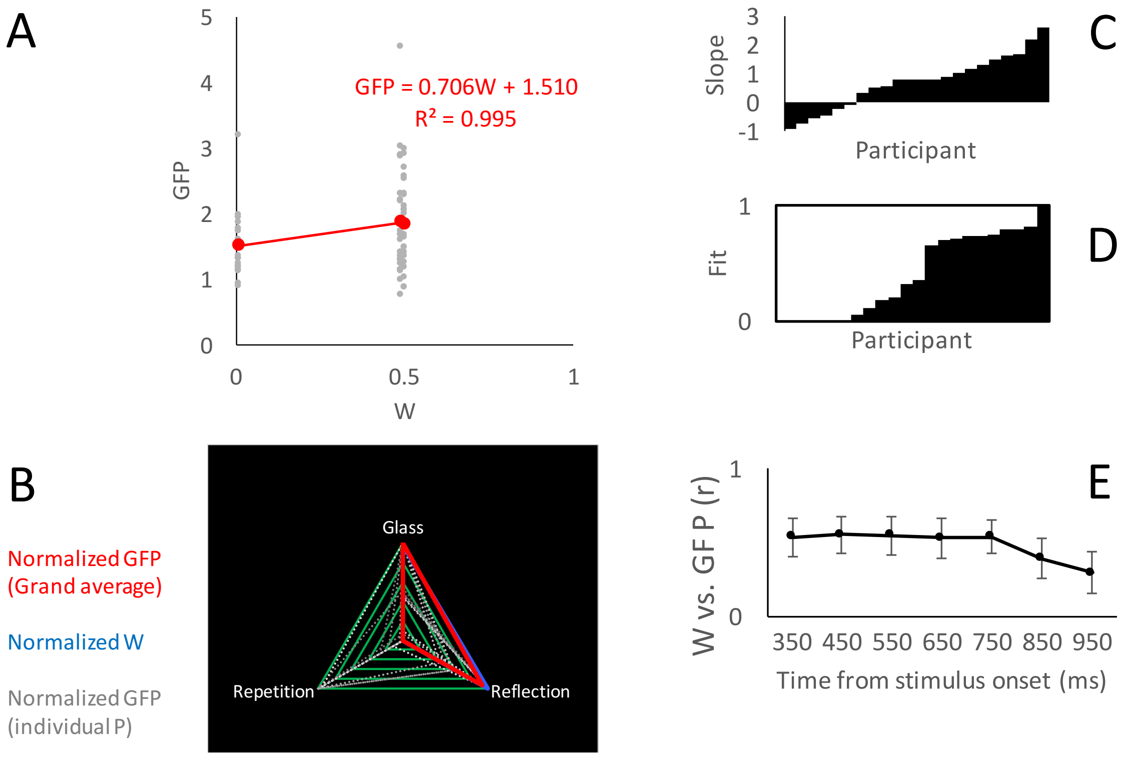

Supplementary Figure 3.1. Global Field Power analysis. Conventions are the same as Supplementary Figure 2.1

Statistical topography analysis

We assume the SPN produced by Glass patterns and reflection had approximately the same topography. Repetition did not generate an SPN, and thus the topographic map was different. Here we confirm this statistically (using the same approach described in Supplementary Materials 2). First, we note that the correlation between the 64 electrodes in the two grand average topographic maps was strong (r = 0.856, Supplementary Figure 3.2A). Next we ran two factor repeated measures ANOVA analysis on the normalized regular-random topographies (300-1000ms). The first factor was Regularity (Glass, Reflection, Repetition), the second was scalp area, with 9 levels (Front left… back right, supplementary Figure 3.2B).

There was a main effect of Area ($F (2.998,62.956) = 11.026$, $p < 0.001$, partial η^2 = 0.344), and borderline Regularity X Area interaction ($F (4.800,100.809) = 2.267$, $p = 0.056$, partial η^2 = 0.097). We next compared pairs of conditions. Importantly, for Glass and reflection, there was no Regularity X Area interaction ($F (3.728, 78.295) = 0.944$, $p = 0.438$, pH0 =0.995). For Glass patterns and repetition there was a Regularity X Area interaction ($F (2.325, 48.830) = 3.113$, $p = 0.046$, partial η^2 = 0.129). There was also borderline Regularity X Area interaction when reflection and repetition were compared ($F (3.163, 66.427) = 2.590$, $p = 0.057$, partial η^2 = 0.110). In summary, Glass patterns and reflection generated a comparable topographic maps with a right lateralized SPN, but repetition did not.

A

	GLASS	REF	REP
**GLASS**			
**REF**	0.856		
**REP**	0.651	0.759	

B

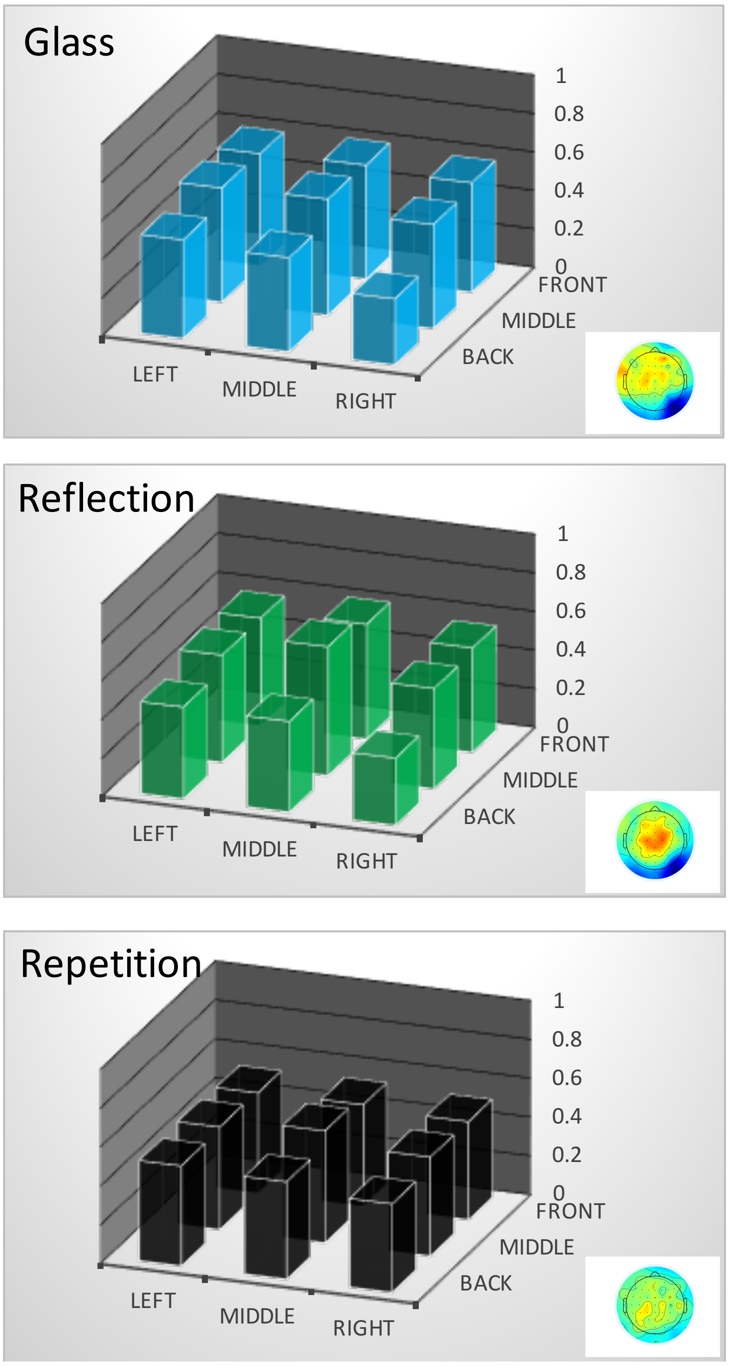

Supplementary Figure 3.2. Statistical topographic analysis of the 300-1000 ms regular – random difference maps. Conventions are the same as Supplementary Figure 2.2

Evolution of the neural symmetry response across the SPN window

Supplementary Figure 3.3 shows topographic difference maps (Regular – Random) in the seven 100 ms sub-intervals across the SPN interval. Although the topographies change over time, there was always a right lateralized posterior for Glass patterns and reflection, but not for repetition. Statistical topography analysis on Glass and reflection conditions found no Regularity X Area interactions at any time point (maximum $F(3.767, 79.097) = 2.211$, $p = 0.079$, $pH0 = 0.991$). This demonstrates that while there was some topographic evolution across the SPN interval, there topographies of Glass and reflection patterns were yoked together. We assume that this is because the brain processed both these regularities in a similar way.

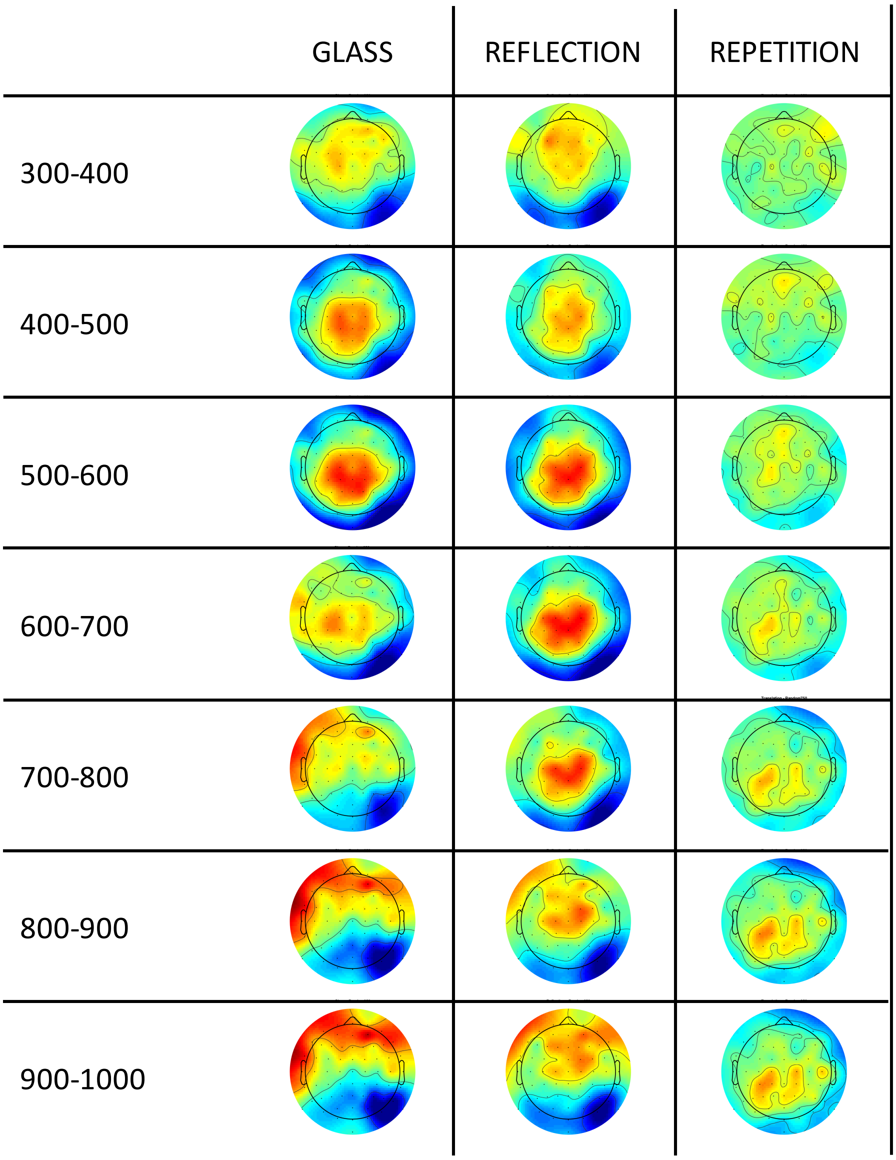

Supplementary Figure 3.3. Sequential topographies across the 300-1000 ms interval. Conventions are the same as Supplementary Figure 2.3

Next we explored the evolution of the fit between W and EEG metrics across the SPN interval. Results are shown in Supplementary Figure 3.4. W was always a significant predictor of SPN at every time window ($p < 0.029$), while W predicted GFP at all windows ($p < 0.024$) except the last one ($p = 0.055$).

Finally, we examined the W vs. SPN correlation coefficients in each window. In all windows mean r was significantly less than 0 ($p < 0.046$). We analysed change over the 7 windows with 1 factor repeated measures ANOVA. There was no main effect of Time on mean correlation coefficient ($F (2.858,60.023) = 2.137$, $p = 0.108$), although the cubic contrast was significant ($F (1,21) = 6.963$, $p = 0.015$, partial $\eta^2 =0.249$). This partly reflects the strong W vs. SPN correlation at 300-400 ms (Figure 9H).

Likewise, at all time windows, the mean W vs. GFP correlations were also significantly less than 0 ($p < 0.048$) There was no main effect of time on W vs. GFP correlations ($F (2.692, 56.540) = 1.028$, $p = 0.381$), despite the decline at the last two time windows (Supplementary Figure 3.1E).

To summarize, in Study 1, we found that the holographic model did a better job predicting SPN and GFP in the first half of the traditional 300-1000 ms SPN window. This change over time was occasionally discernible in Study 2, but it was much less robust, and not found across all analyses. The most basic result in Study 2 that W explained most variance in SPN and GFP throughout the SPN window.

300 – 400
SPN (µV) = -3.313W -0.240
$\chi 2$ = 20.145, $p < 0.001$
R^2 = 0.933

GFP (µV) = 0.864W +1.307
$\chi 2$ = 13.202, $p < 0.001$
R^2 = 0.998

400 – 500
SPN (µV) = -1.528W-0.423
$\chi 2$ = 4.825 p = 0.028
R^2 = 0.975

GFP (µV) = 0.707W+1.442
$\chi 2$ =10.964, $p < 0.001$
R^2 = 0.852

500 – 600
SPN (µV) = -2.239W-0.508
$\chi 2$ = 7.063, p = 0.008
R^2 = 0.995

GFP (µV) = 1.246W + 1.538
$\chi 2$ =16.014, $p < 0.001$
R^2 = 0.998

600 – 700
SPN (µV) = -2.800W-0.452
$\chi 2$ = 8.605, p = 0.003
R^2 = 0.985

GFP (µV) = 1.288W + 1.653
$\chi 2$ = 14.435, $p < 0.001$
R^2 = 0.980

700 – 800
SPN (µV) = -3.384W -0.209
$\chi 2$ = 9.291, p = 0.002
R^2 = 0.992

GFP (µV) = 1.103W + 1.748
$\chi 2$ =9.077, p = 0.003
R^2 = 0.994

800 – 900
SPN (µV) = -3.928W -0.293
$\chi 2$ = 9.700, p = 0.002
R^2 = 0.996

GFP (µV) = 0.915W + 1.945
$\chi 2$ =5.145, p = 0.023
R^2 = 0.812

900 – 1000
SPN (µV) = -4.200-0.347
$\chi 2$ = 8.580, p = 0.003
R^2 > 0.999

GFP (µV) = 0.910W+2.049
$\chi 2$ =3.685, p = 0.055
R^2 = 0.741

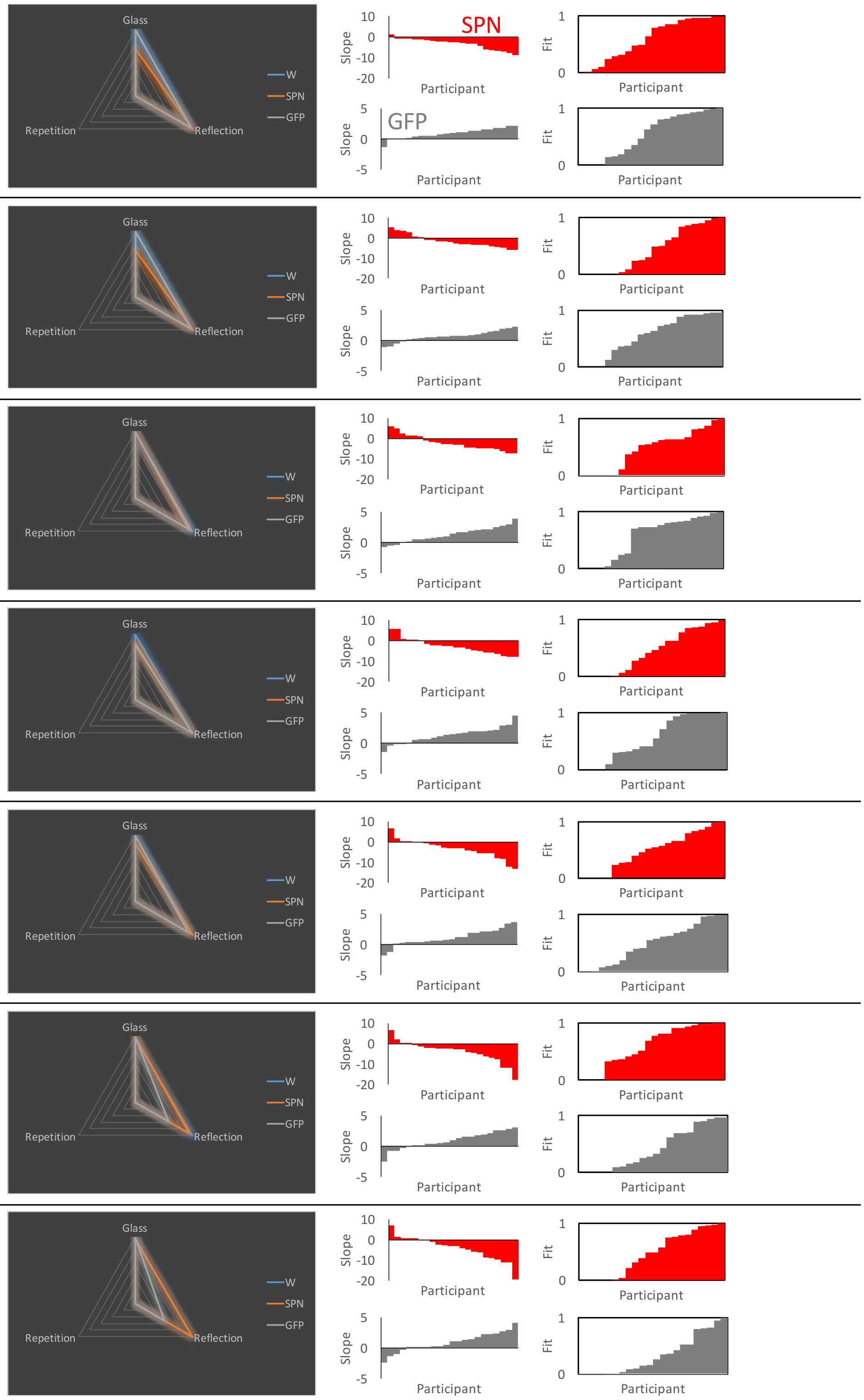

Supplementary Figure 3.4. W vs. SPN relationship across the 300-1000 ms interval. Conventions are the same as Supplementary Figure 2.4
