# Supplementary material for: An Electrophysiological Index of Perceptual Goodness
Source: Cereb Cortex. 2016 Dec 26;26(12):4416–34. doi: 10.1093/cercor/bhw255 (PMC5193141; doi:10.1093/cercor/bhw255)
Supplement: Supplementary Data [file supp_bhw255_Supplementary_Materials4.pdf]

Supplementary Materials 4: Extra information for study 3

EEG Method

As with studies 1 and 2, participants were required to fixate during 1.5 second baseline and 1.5 second presentation periods, and judgments were reported after stimulus offset. Response protocol was the same as other EEG experiments. Mean error rate was 15% in the 1-fold condition, and 1-3% for multiple reflections and in the random condition (excluding one anomalous participant who pressed 'Reflection' on most trials). An average of 11.41 ICA components was removed from each participant (min = 3, max =24). The mean trial exclusion rate was 6.8%, and was similar between conditions (min 6.1%, max 7.7%).

Effect of pattern segmentation in the random condition

Random patterns were constructed using the same algorithm as the reflections, and again there were 1-5 implicit folds, albeit with random positioning on either side of the axes. In the main analysis we averaged all random trials together to produce a single random ERP (black line in Supplementary Figure 4.1A). Here we test whether this simplification is warranted by examining the random 1 to 5 fold conditions.

Compared to the difference between reflection conditions (Supplementary Figure 4.1A), the difference in the random conditions is small (Supplementary Figure 4.1B). We analysed the random data with the same repeated measures ANOVA we used for the SPN [Time window, (early, late) X Folds (1:5)]. There was no Time X Folds interaction ($F(4,80) = 0.612$, $p = 0.655$). However, there was a weak main effect of Folds ($F(4, 80) = 2.643$, $p = 0.040$, partial $\eta^2 = 0.117$), primarily because amplitude lower in the 1F random patterns. This relatively small effect was not predicted a-priori could be a false positive. However, it could result some residual perceptual regularity in the 1F random patterns. This could be

from accidental pairing of the randomly positioned elements (see Supplementary materials 1) combined with the cardinal orientation of salient sub-groups (absent in the 3 and 5-Fold random patterns at least).

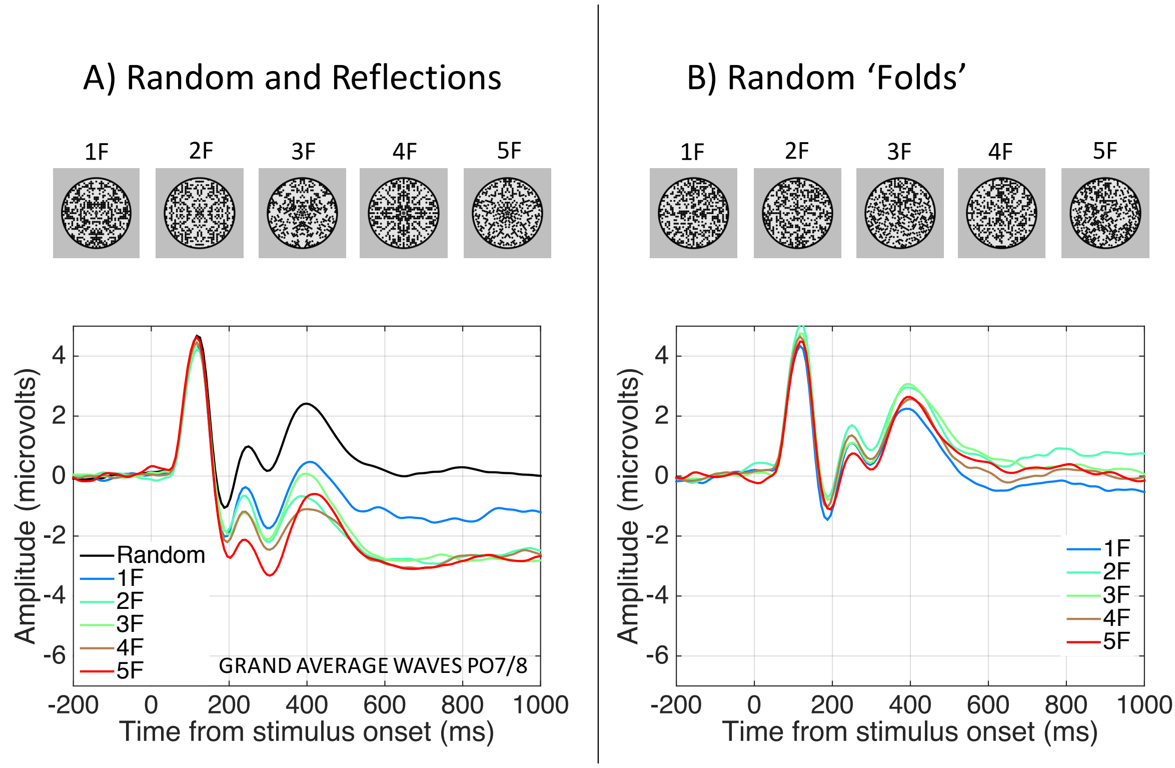

Supplementary Figure 4.1. ERPs produced by random and reflection patterns with 1-5 folds. A) ERPs from reflection conditions and the average random wave. B) ERPs from 1-5 fold random patterns. Example stimuli are show above.

Global Field Power

GFP results are shown in Supplementary Figure 4.2. As with the SPN analysis, we considered two time windows, 350-450ms and 600-1000 ms. We followed the same analysis steps used for SPN. First analysed GFP with two factor repeated measure ANOVA (Window (350-450, 600-1000) X Folds (1-5)).

There was a main effect of Folds ($F(4, 84) = 6.117$, $p < 0.001$, partial $\eta^2 = 0.226$), but there was no Window X Folds interaction ($F(2.389, 50.171) = 1.961$, $p = 0.144$). The lack of interaction suggests there is no difference between GFP in the early and late windows. This

is an important departure from the SPN results, where there was a clear change between early and late windows. However, we ran separate linear regression analyses in early and late to facilitate comparison with SPN effects (while cautioning that the early and late windows were not designed for GFP analysis, and were applied post hoc based on SPN waveforms at PO7/8).

Normalized GFP, W and T are overlaid in the radar plots line graphs in Supplementary Figure 4.2. In the early window, grand average GFP is closer to W (R^2 = 0.814) than T (R^2 = 0.419). Linear mixed effects analysis found that GFP was related to W (GFP μV = 1.377W, χ^2 (1) = 10.226, p = 0.001) and less so to T (GFP μV = 0.875T, χ^2 (1) = 5.733, p = 0.017). Indeed, analysis of residuals found that W captured more variance in GFP than T in this early interval (χ^2 (1) = 12.538, p < 0.001).

In the late window (600-1000 ms), W and T explained approximately the same amount of variance in GFP (R^2 = 0.677 vs. 0.617). Linear mixed effects analysis found that both were significant predictors of GFP in the late window (GFP μV = 1.189W, (χ^2 (1) = 9.779, p = 0.002, GFP μV = 1.006T (χ^2 (1) = 9.829, p = 0.002). Analysis of residuals found no advantage for T over W or vice versa (χ^2 (1) = 1.310, p = 0.252).

We also analysed the correlation coefficients with a 2 x 2 repeated measures ANOVA [2 Metric (T, W) X 2 Window (Early, Late)]. There was a Metric X Window interaction (F (1,21) = 22.933, p < 0.001, partial η^2 = 0.522). GFP was more strongly correlated with W than T In the early window (t (21) = 3.296, p = 0.003). There was no significant difference in the late window (t (21) = -0.724, p = 0.477).

In summary, GFP is generally more closely related to W than T in the early window (350-450 ms), while GFP was not very similar to either W or T in the late window. This broadly parallels analysis of the SPN regarding the early window.

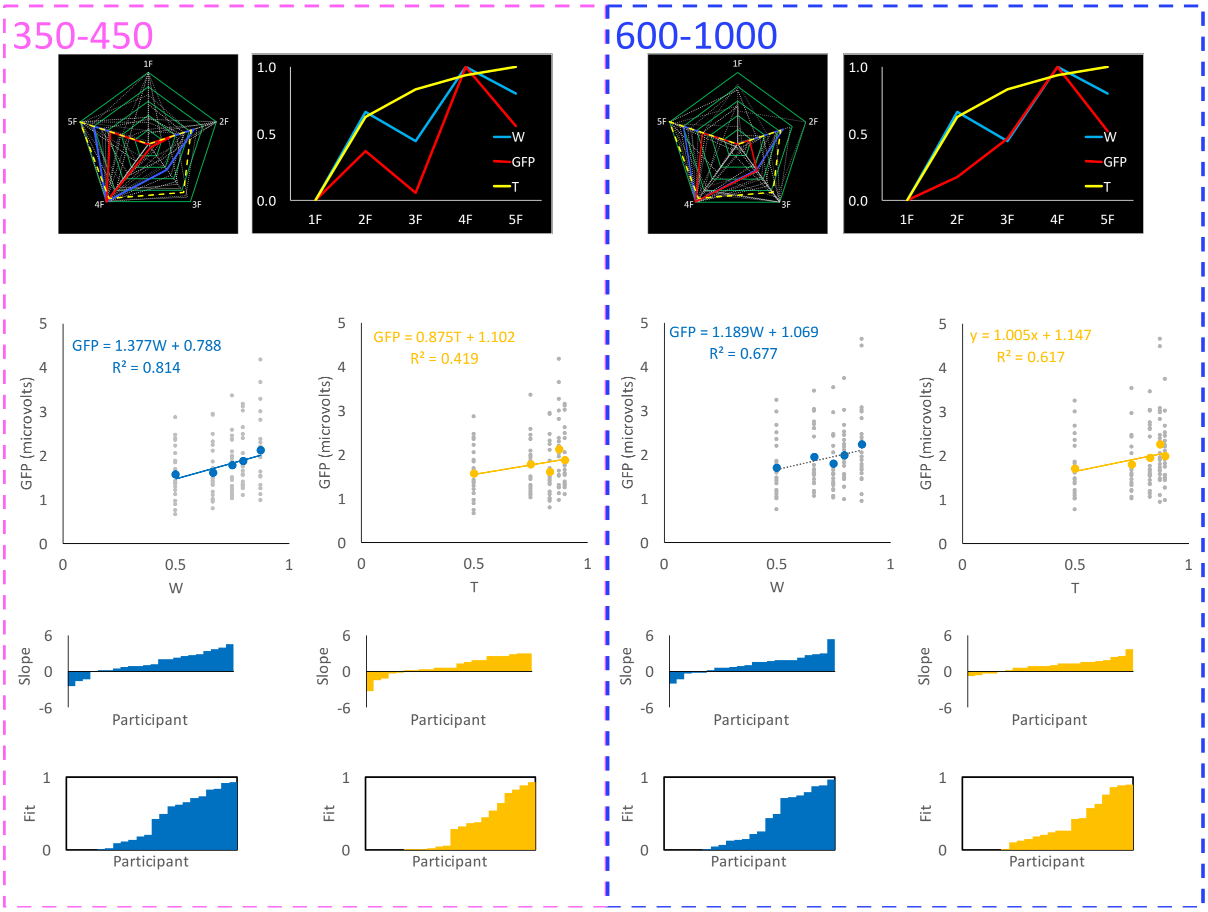

Supplementary Figure 4.2. Global Field Power analysis.

Statistical Topography Analysis

We took the same approach to statistical topography analysis as in Studies 1 and 2. The correlation matrix in Supplementary Figure 4.3 shows a minimum correlation of r = 0.88. This suggests that amplitude across the 64 electrodes was distributed in a similar way in all 5 conditions. Second, we analysed normalized topographies with 2 factor repeated measures ANOVA [5 folds (1F 2F 3F 4F 5F) X 9 area (Front Left … Back right)]. The 9 electrode clusters were the same as used in previous analysis. The SPN appears as shorter columns at the back clusters in Supplementary Figure 4.3. There was a strong main effect of Area (F (3.021, 63.439) = 31.243, p <0.001, partial η^2 = 0.598), but importantly, there was *no* Folds X Area interaction (F (32,672), = 1.201, p = 0.199, pH0 > 0.999).

	1F	2F	3F	4F	5F
**1F**					
**2F**	0.905				
**3F**	0.904	0.979			
**4F**	0.877	0.972	0.974		
**5F**	0.902	0.960	0.970	0.967	

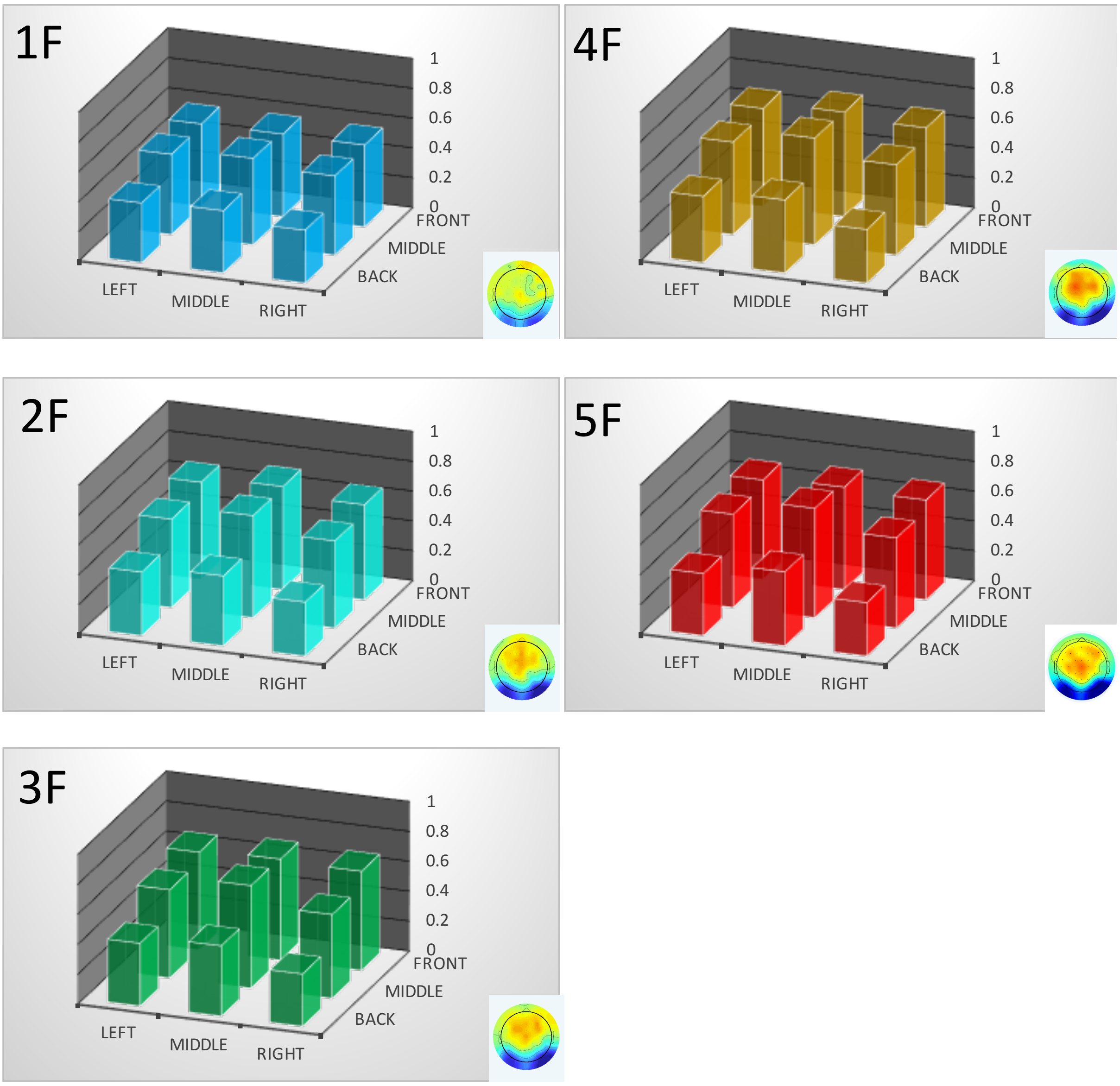

Supplementary Figure 4.3. Statistical topographic analysis of the 300-100 ms regular – random difference maps

We caution that we assumed sphericity for the interaction. We could not compute the Mauchly's W statistic because the ratio of conditions / participants was too great. However, this *increases* the chances of a Type 1 error, and can only work against our assumption of topographic invariance.

Evolution of the neural symmetry response across the SPN window

We ran further analysis on development of the SPN and GFP signal during the 300 to 1000 ms window. As with studies 1 and 2, we examined EEG data in seven consecutive 100 ms sub windows. We caution that this analysis less informative than for studies 1 and 2, given that we already found that W was a better predictor of SPN and GFP at an early time window (350-450 ms) and T was a better predictor of SPN at a later window (600-1000 ms). However, we report analysis of the sub-windows for consistency and to facilitate comparison with the other studies.

Supplementary Figure 4.4 shows topographic difference maps in the seven 100 ms sub-intervals. Statistical topography analysis found no Folds X Area interaction at 300-400 ms ($F(32,672) = 1.367$, $p = 0.087$, $pH0 > 0.999$) or 400-500 ms ($F(32,672) = 1.180$, $p = 0.230$, $pH0 > 0.999$). This suggests that in the earlier intervals where SPN and GFP were closely related to W, we can certainly assume topographic invariance at this point.

However, there were some potential topographic differences at 500-600 ms ($F(32,672) = 1.565$, $p = 0.026$, partial $\eta^2 = 0.069$) and in the last three windows (minimum effect, $F(32,672) = 1.725$, $p = 0.008$, partial $\eta^2 = 0.076$). Despite this, we believe the assumption of topographic invariance is valid at late time windows as well. First, sphericity was assumed for this interaction, which increases Type 1 error rate. Second, we are conducting 7 multiple analyses, and inflating type 1 error rate further. Third, the effect size

of the Folds X Area interactions was small (*maximum* effect F (32,672) = 2.409, $p < 0.001$, partial $\eta^2 = 0.103$). In contrast, the main effect of Area was large at all time windows (*minimum* effect F (3.262, 68.498) = 18.737, $p < 0.001$ partial $\eta^2 = 0.472$). We can thus be confident our Folds manipulation mainly had an effect of SPN amplitude, not topography, and that this was true throughout the SPN interval.

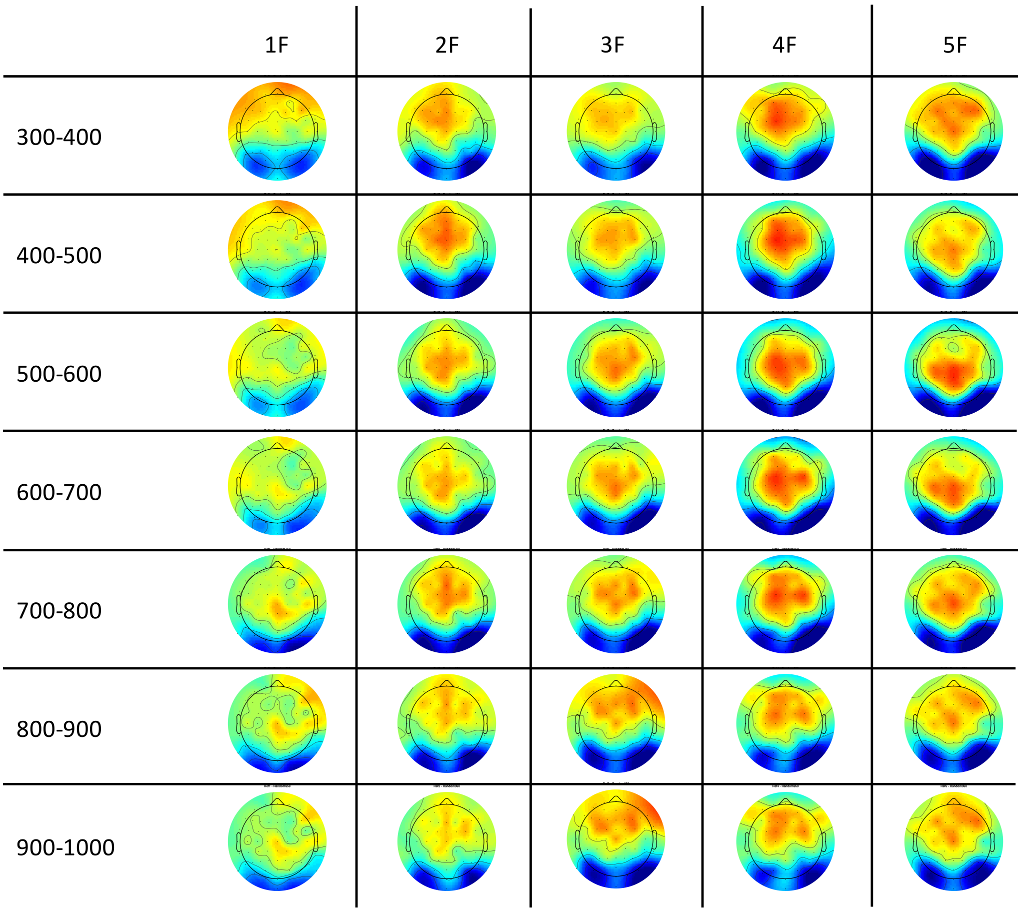

Supplementary Figure 4.4. Sequential topographies across the 300-1000 ms interval.

We next considered evolution of the fit between W, T, SPN and GFP metrics over the time. Results are shown in Supplementary Figure 4.5 (for W) and Supplementary Figure 4.6 (for T). Linear mixed effects analysis is shown in the left columns: It can be seen that W and T are both significant predictors of SPN and GFP at every time window. There was a

discernible reduction in the predictive power of W across intervals, and an increase in T. As usual, this can be most easily visualized in the radar plots.

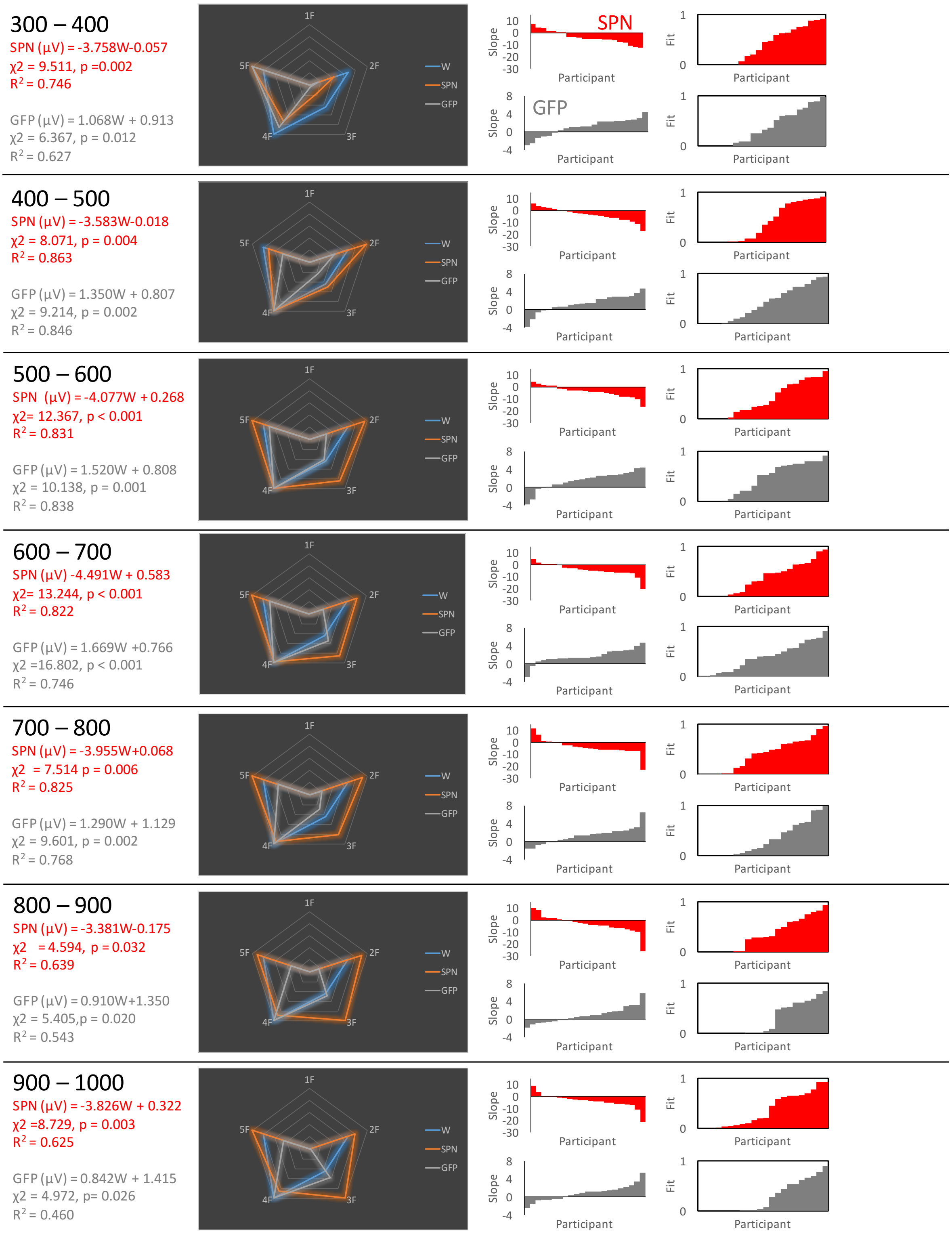

Supplementary Figure 4.5. W vs. SPN relationship across the 300-1000 ms interval.

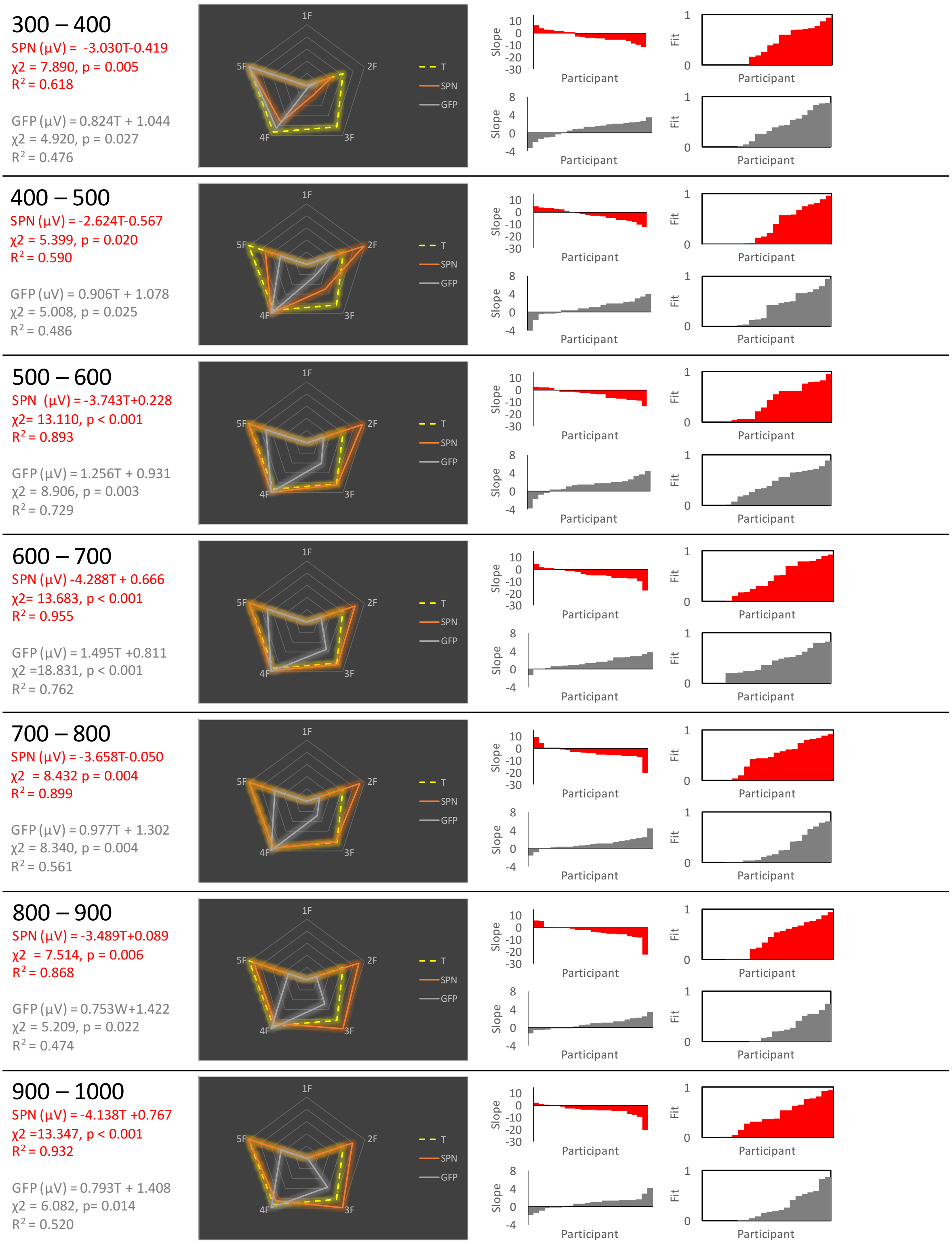

Supplementary Figure 4.6. T vs. SPN relationship across the 300-1000 ms interval.

Analysis of residuals was used to compare the holographic and transformational models statistically. At 400-500 ms, W predicted more variance in SPN, and also more variance in GFP (Supplementary Table 4.1). At later time windows T predicted more variance than W.

Supplementary Table 4.1. Linear mixed effects analysis of W and T at successive time windows. Significant effects are shown in bold.

SPN

	**W**			**T**			**Comparison**		
**Time**	**Slope**	**χ^2 (1)**	**p**	**Slope**	**χ^2 (1)**	**p**		**χ^2 (1)**	**p**
**350**	-3.758	9.511	**0.002**	-3.030	7.890	**0.005**	W > T	3.787	0.052
**450**	-3.583	8.071	**0.004**	-2.624	5.399	**0.020**	W > T	5.821	**0.016**
**550**	-4.077	12.367	**0.000**	-3.743	13.110	**0.000**	T > W	2.602	0.107
**650**	-4.491	13.244	**0.000**	-4.288	13.683	**0.000**	T > W	4.146	**0.042**
**750**	-3.955	7.514	**0.006**	-3.658	8.432	**0.004**	T > W	2.353	0.125
**850**	-3.381	4.594	**0.032**	-3.489	7.514	**0.006**	T > W	4.613	**0.032**
**950**	-3.826	8.729	**0.003**	-4.138	13.347	**0.000**	T > W	6.989	**0.008**

GFP

	**W**			**T**			**Comparison**		
**Time**	**Slope**	**χ^2 (1)**	**p**	**Slope**	**χ^2 (1)**	**p**		**χ^2 (1)**	**p**
**350**	1.068	6.367	**0.012**	0.824	4.920	**0.027**	W > T	3.472	0.062
**450**	1.350	9.214	**0.002**	0.906	5.008	**0.025**	W > T	8.924	**0.003**
**550**	1.520	10.138	**0.001**	1.256	8.906	**0.003**	W > T	2.657	0.103
**650**	1.669	16.802	**0.000**	1.495	18.831	**0.000**	T > W	1.712	0.191
**750**	1.290	9.601	**0.002**	0.977	8.340	**0.004**	W > T	3.439	0.064
**850**	0.910	5.405	**0.020**	0.753	5.209	**0.022**	W > T	0.636	0.425
**950**	0.842	4.972	**0.026**	0.793	6.082	**0.014**	T > W	0.805	0.370

Patterns in individual participant SPN correlations are shown Supplementary Figure 4.7A. This data was analysed with a repeated measures ANOVA [2 Model (W, T) X 7 Time window}. There was no main effect of Model (F (1,21) = 0.250, p = 0.622) or Time (F (2.112, 44.350) = 0.853, p = 0.439). There was a significant Model X Time interaction (F(4.236,

88.951) = 4.334, $p = 0.003$, partial η^2 0.171). However, there were no significant effects of time when W and T correlations were analysed separately (F (2.331, 48.954) = 0.463, p = 0.661; F (2.029, 42.605) = 1.426, p = 0.252) and the difference between W and T correlations did not reach significance at any time point ($p > 0.084$). However, all mean correlations shown Supplementary Figure 4.7A were significantly < 0 ($p < 0.009$)

Patterns in individual GFP correlations are shown in Supplementary Figure 4.7B. Again there was a Model X Time interaction (F (4.032, 84.670) = 3. 986, $p = 0.005$, partial η^2 0.160). The apparent peak correlation at 600-700 ms produced a significant quadratic contrast for both W (F (1,21) = 5.133, $p = 0.034$, , partial η^2 0.196) and T (F (1,21) = 5.300, p = 0.032, partial η^2 0.202). Pairwise comparisons revealed that W correlation was greater at 400-500 ms only (t (21) = 2.378, $p = 0.027$). The mean W vs. GFP correlation was significant ($p < 0.015$) at all windows apart from the last two ($p > 0.065$). The mean T vs. GFP correlation was significantly < 0 at all time windows ($p < 0.045$).

In summary, the analysis of sub-windows confirmed that W is a better predictor of SPN at early time points, and T is a better predictor at later time points. However, the windows were chosen for consistency with analysis of studies 1 and 2, and are not optimized to distinguish between holographic and transformational models. As we report in the manuscript, the holographic model explains most variance in SPN amplitude at 350-450 ms, where the predicted dip at 3 and 5-fold symmetry was clear, and there was a distinct peak in the ERP waveforms.

A) Sustained Posterior Negativity (SPN)

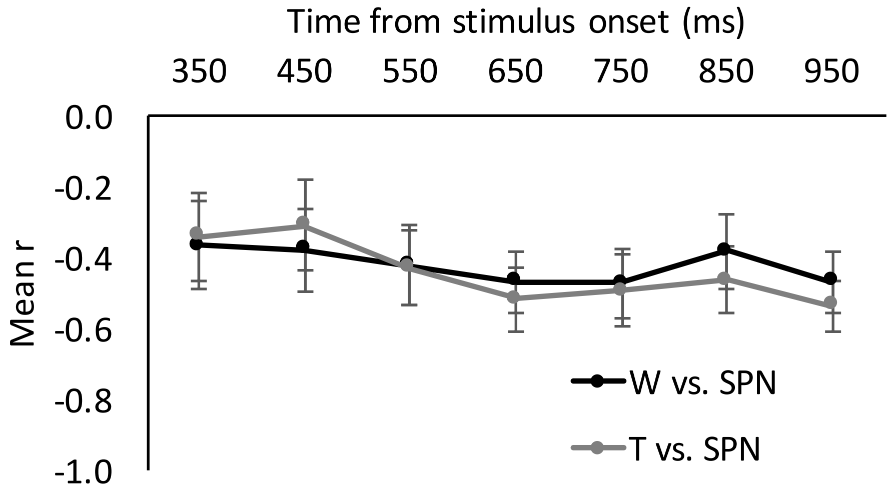

B) Global Field Power (GFP)

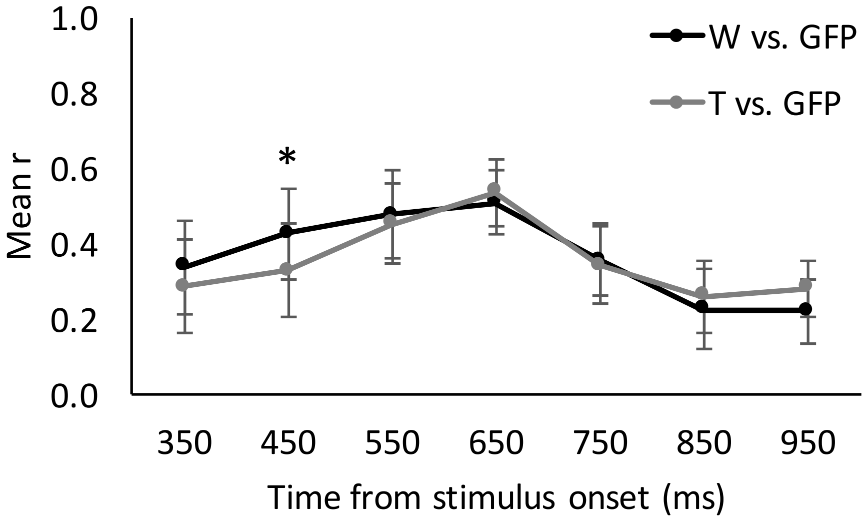

Supplementary Figure 4.7. T and W correlations over the 300-1000 ms interval. A) Mean correlation between goodness metrics and SPN amplitude in seven successive 100 ms time bins. B) GFP data analysed in the same way. (* < 0.05, here the mean W vs. GFP correlation was higher than mean T vs. GFP correlation). Error bars +/- 1 S.E.M.
