# Supplementary material for: An Electrophysiological Index of Perceptual Goodness
Source: Cereb Cortex. 2016 Dec 26;26(12):4416–34. doi: 10.1093/cercor/bhw255 (PMC5193141; doi:10.1093/cercor/bhw255)
Supplement: Supplementary Data [file supp_bhw255_Supplementary_Materials5.pdf]

Supplementary Materials 5: Extra information for study 4

EEG Method

As with studies 1, 2 and 3 participants were required to fixate throughout the 1.5 second baseline and 1.5 second stimulus intervals. Judgments were reported after stimulus offset. Response protocol the same as our other EEG experiments. In the 1-Fold Experiment, mean error rate was 3% in the symmetry condition, 11% in the anti-symmetry condition (excluding 1 participant who consistently misreported anti-symmetry as random) and 5% in the random condition. On average of 7.91 ICA components were removed from each participant (min = 3, max = 15). The mean trial exclusion rate was 6.5%, and was similar between conditions (min 6.1%, max 6.7%). In the 4-Fold experiment, Mean error rate was 2% for symmetry, 7% for anti-symmetry, and 5% for random. On average 8.14% ICA components were removed from each participant (min 2, max 21). Trial exclusion rate was 8.4%, and similar in all conditions (8.1 – 9%).

Global Field Power

Like the SPN, GFP was analysed with mixed ANOVA. There was one within-participants Factor [Regularity type (Symmetry, Anti-Symmetry)] and one between-participants Factor [Folds, (1,4)]. Results are shown in Supplementary Figure 5.1. GFP was significantly greater in the 4-Fold experiment than the 1-Fold experiment (F (1,42) 6.686, $p = 0.013$, partial $\eta^2 = 0.137$). GFP was also significantly greater in the Symmetry condition than in the Anti-symmetry condition (F (1,42) = 12.548, $p = 0.001$, partial $\eta^2 = 0.230$). There was no Folds X Regularity type interaction (F (1,42) = 1.428, $p = 0.239$). Nevertheless, we explore results from 1-fold and 4-fold experiments separately. In the 1-Fold experiment, GFP was higher in

the symmetry than anti-symmetry condition (t (21) = 3.194, p = 0.004). This effect did not reach significance in the 4-Fold experiment (t (21) = 1.749, p = 0.095).

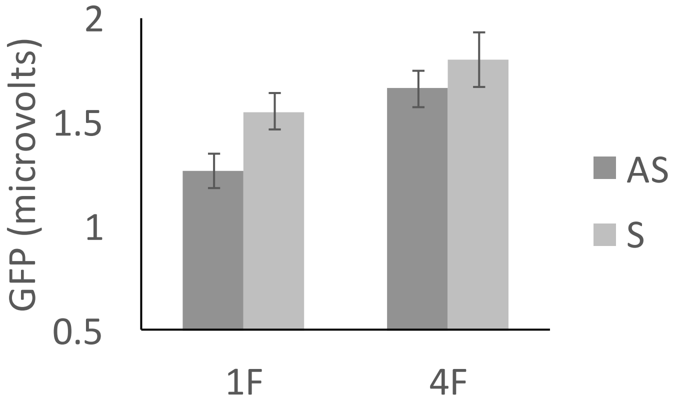

Supplementary Figure 5.1. Global Field Power analysis. Mean GFP in the Symmetry and anti-symmetry conditions of the 1F and 4F experiments. Error bars = +/- 1 S.E.M.

Statistical Topography Analysis

Statistical topography analysis was the same as in the previous experiments. This confirmed that SPN topography was similar in all four conditions. Normalized topographic difference maps (regular- random, 300-1000 ms) were first analysed with mixed ANOVA. If the assumption of topographic invariance is correct, then there should be no interactions involving Area. Data is shown in Supplementary Figure 5.2. As usual, the SPN manifests here as shorter columns at the back.

Unsurprisingly, there was a main effect of Area (F (4.480, 188.141) 50.467, $p < 0.001$, partial $\eta^2 = 0.546$). There was also a Folds X Area interaction (F (4.480, 188.141) = 10.309, $p < 0.001$, partial $\eta^2 = 0.197$). This means we cannot assume the SPN topography was identical in 1-Fold and 4-Fold experiments. This is a between-participants factor, so we might expect topographic differences.

Importantly, there was no Regularity X Area interaction (F (4.015, 168.630) = 1.591, 0.179, pH0 = 0.997), and no Regularity X Area X Folds interaction (F (4.015, 168.630) = 0.812, p = 0.519, pH0 = 0.998). Furthermore, there was no Regularity X Area interaction in the 1-Fold experiment (F (3.772, 79.205) = 1.398, p = 0.244, pH0 = 0.994) or in the 4-Fold experiment (F (3.782, 79.426) = 0.910, p = 0.458, pH0 = 0.995). This again confirms that Symmetry and Anti-symmetry produced ERPs with essentially the same topography.

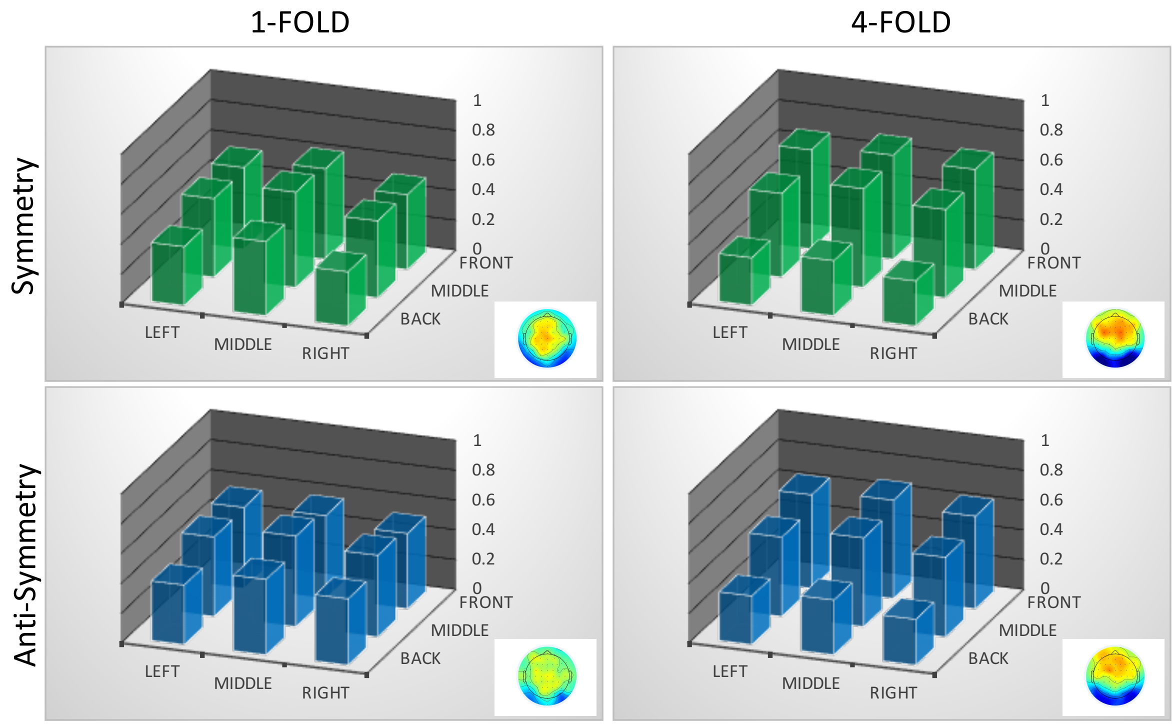

Supplementary Figure 5.2. Statistical topographic analysis of the 300-100 ms regular – random difference maps.

Evolution of the neural symmetry response across the SPN window.

Again we considered SPN data from seven consecutive 100 ms sub windows [(300 – 400), (400 – 500), (500 – 600), (600 – 700), (700 – 800), (800-900) and (900-1000 ms)]. Topographic difference maps each sub-window are shown in Supplementary Figure 5.3. Although topography clearly evolves across the SPN interval, the same basic

ordering remains, so 4-Fold patterns produced a larger SPN than 1-Fold, and Symmetry produced a slightly larger SPN than anti-symmetry.

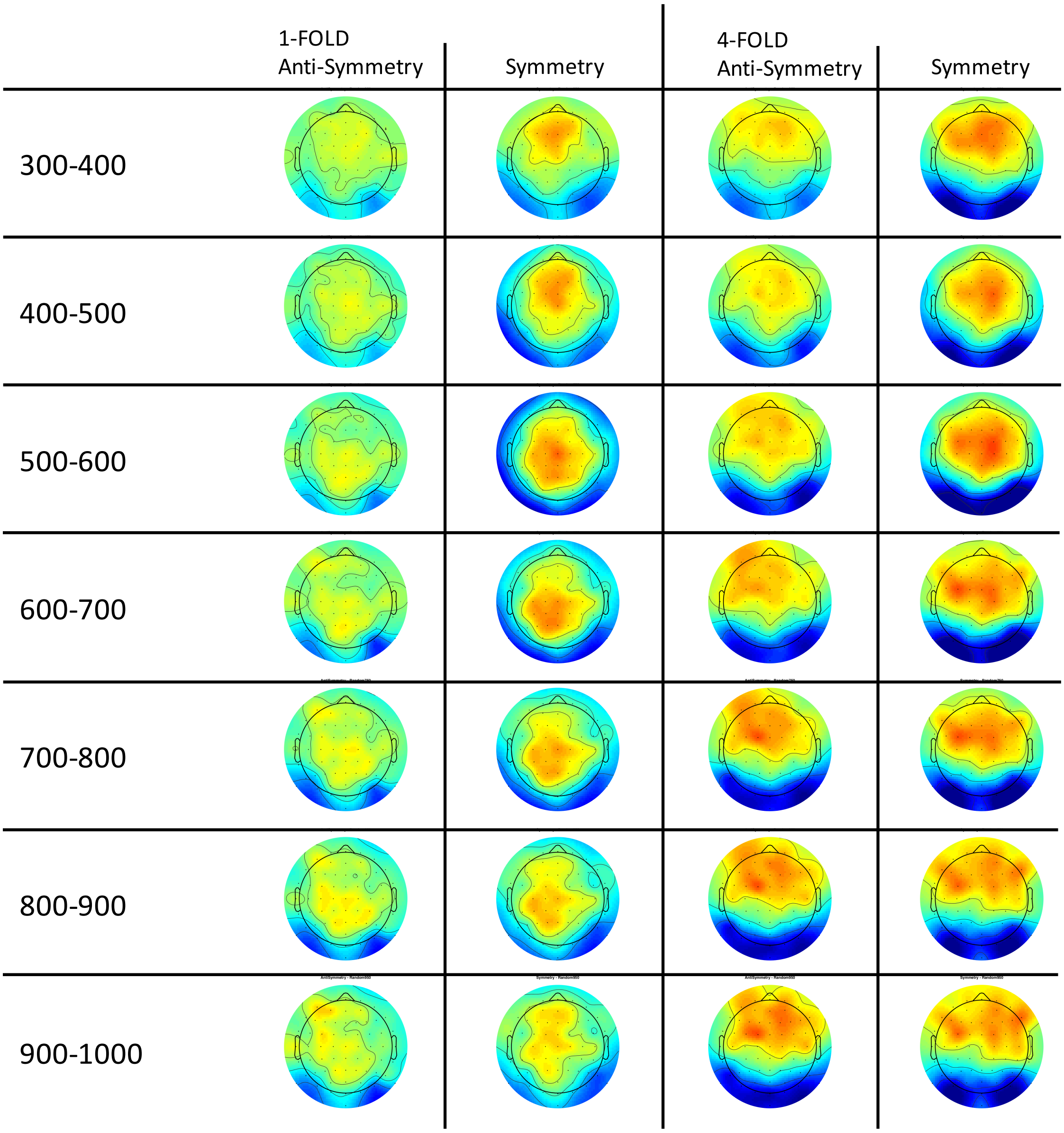

Supplementary Figure 5.3. Sequential topographies across the 300-1000 ms interval.

Statistical topography analysis was applied to each window. We analysed the 1 and 4 fold experiments separately, because were most interested in topography of symmetry and anti-symmetry (rather than the topography of 1 and 4-Fold patterns

recorded from different groups of participants). Of the 14 Regularity X Area interactions examined, there was only 1 weak effect (In the 4-Fold 300-400 ms window, $F(3.762, 79.002) = 2.901$, $p = 0.030$, partial $\eta^2 = 0.121$). This contrasts with a ubiquitous and strong main effect of Area (minimum effect in 1-Fold 500-600 ms window, $F(3.737, 78.470) = 6.144$, $p < 0.001$, partial $\eta^2 = 0.226$). We can safely conclude that the topography of symmetry and anti-symmetry ERPs remained comparable to each other across the SPN window. Symmetry and anti-symmetry are thus likely to activate the same extrastriate network.
